# Lifecycle of dynamic covalent polar-olefin macrocycles via entropy-driven ring-opening polymerization and closed-loop chemical recycling

**DOI:** 10.1093/nsr/nwaf484

**Published:** 2025-11-06

**Authors:** Pengyun Li, Chong Li, Mengying Lei, Ruirui Gu, He Tian, Da-Hui Qu

**Affiliations:** Key Laboratory for Advanced Materials and Joint International Research Laboratory of Precision Chemistry and Molecular Engineering, Feringa Nobel Prize Scientist Joint Research Center, Frontiers Science Center for Materiobiology and Dynamic Chemistry, Institute of Fine Chemicals, School of Chemistry and Molecular Engineering, East China University of Science and Technology, Shanghai 200237, China; Key Laboratory for Advanced Materials and Joint International Research Laboratory of Precision Chemistry and Molecular Engineering, Feringa Nobel Prize Scientist Joint Research Center, Frontiers Science Center for Materiobiology and Dynamic Chemistry, Institute of Fine Chemicals, School of Chemistry and Molecular Engineering, East China University of Science and Technology, Shanghai 200237, China; Key Laboratory for Advanced Materials and Joint International Research Laboratory of Precision Chemistry and Molecular Engineering, Feringa Nobel Prize Scientist Joint Research Center, Frontiers Science Center for Materiobiology and Dynamic Chemistry, Institute of Fine Chemicals, School of Chemistry and Molecular Engineering, East China University of Science and Technology, Shanghai 200237, China; Key Laboratory for Advanced Materials and Joint International Research Laboratory of Precision Chemistry and Molecular Engineering, Feringa Nobel Prize Scientist Joint Research Center, Frontiers Science Center for Materiobiology and Dynamic Chemistry, Institute of Fine Chemicals, School of Chemistry and Molecular Engineering, East China University of Science and Technology, Shanghai 200237, China; Key Laboratory for Advanced Materials and Joint International Research Laboratory of Precision Chemistry and Molecular Engineering, Feringa Nobel Prize Scientist Joint Research Center, Frontiers Science Center for Materiobiology and Dynamic Chemistry, Institute of Fine Chemicals, School of Chemistry and Molecular Engineering, East China University of Science and Technology, Shanghai 200237, China; Key Laboratory for Advanced Materials and Joint International Research Laboratory of Precision Chemistry and Molecular Engineering, Feringa Nobel Prize Scientist Joint Research Center, Frontiers Science Center for Materiobiology and Dynamic Chemistry, Institute of Fine Chemicals, School of Chemistry and Molecular Engineering, East China University of Science and Technology, Shanghai 200237, China

**Keywords:** dynamic covalent chemistry, polar olefin bonds, entropy-driven ring-opening polymerization, closed-loop chemical recycling

## Abstract

The global plastic pollution crisis urgently demands closed-loop chemical recycling strategies. While recyclable polymers via olefin metathesis have been widely explored, the development of metal-free methods operating under mild conditions remains a significant challenge. Here, we present the lifecycle design of polar-olefin-derived macrocycles as novel monomers capable of undergoing reversible entropy-driven ring-opening polymerization (ED-ROP) through organic base-catalyzed metathesis of polar olefin bonds. High-molecular-weight polymers were efficiently produced via bulk melt polymerization. Kinetic studies and mass analyses indicated the formation of cyclic polymer topologies through insertion and ring expansion, with polymerization thermodynamically driven by an increase in conformational entropy. By shifting the equilibrium of polar-olefin metathesis in dilute solution, these polymers enable efficient closed-loop depolymerization and monomer recovery. This approach establishes a versatile platform based on polar olefin chemistry, advancing the design of recyclable materials with tailored dynamic functionalities.

## Teaser

Bio-based macrocycles bearing dynamic polar-olefin bonds undergo entropy-driven ring-opening polymerization to yield cyclic polymers that can be depolymerized on demand, offering a practical route to circular plastics.

## INTRODUCTION

Over the past few decades, synthetic polymers have revolutionized modern industry and daily life due to their superior performance and processability. Global annual polymer production now exceeds 400 million metric tons and continues to grow steadily [1]. However, the lack of sustainable and cost-effective end-of-life plastic management strategies has exacerbated the global plastic waste crisis [2]. Consequently, researchers are not only advancing efficient recycling techniques for conventional polymers but also designing inherently recyclable synthetic polymers from the bottom up to enable a sustainable circular economy [3–9]. The emergence of dynamic covalent chemistry (DCC) has unlocked novel pathways for polymer circularity. By integrating DCC into polymer synthesis, closed-loop monomer recycling becomes feasible, wherein polymers can be selectively depolymerized back into their original monomers and subsequently repolymerized, thereby circumventing the performance degradation commonly associated with traditional recycling methods [9–14].

Recent advances in DCC-based lifecycle design have demonstrated that ring-opening polymerization (ROP) of cyclic monomers, followed by closed-loop depolymerization, represents a particularly promising chemical recycling strategy that effectively prevents byproduct formation during polymerization while enabling precise molecular weight control. Extensive studies have focused on ROP of small cyclic monomers (3- to 8-membered rings) containing dynamic covalent bonds, including lactones [15–17], thiolactones [18–20], acetals [21,22], amides [23,24], carbonates [25] and 1,2-dithiolanes [26,27], which undergo subsequent efficient depolymerization. These polymerizations are typically driven by ring-strain release, resulting in a negative polymerization enthalpy (Δ*H* < 0) [28]. Through rational design of monomer structures and catalytic systems, researchers have achieved fine modulation of ring-strain, facilitating efficient depolymerization and repolymerization within optimal temperature windows, thereby establishing sustainable lifecycles for polymeric materials [29,30].

In contrast, large-ring monomers exhibit minimal ring strain and thus minimal enthalpy changes during ring opening. Their polymerization is driven primarily by the gain in conformational entropy (Δ*S* > 0), a process known as entropy-driven ROP (ED-ROP) [31]. According to the Gibbs free energy relation (Δ*G* = Δ*H* − *T*Δ*S*), when both Δ*H* > 0 and Δ*S* > 0, polymerization becomes thermodynamically favorable only above a characteristic floor temperature (*T*_f_), with elevated temperatures favoring polymer formation. Monomer concentration critically influences the polymerization equilibrium via entropy modulation: high concentrations enhance conformational entropy gains, favoring polymerization, while dilute conditions shift the equilibrium toward depolymerization due to dominant translational entropy [32]. Typically, this strategy enables the fabrication of *T*_f_-regulated polymers that outperform ROP-based ceiling temperature (*T*_c_)-regulated systems by permitting polymerization within an industrially feasible temperature window, providing enhanced thermodynamic stability at elevated temperatures without requiring catalyst removal, and enabling low-energy depolymerization at near-ambient temperatures [33]. Recent advances demonstrate ED-ROP in large-ring monomers incorporating diverse dynamic motifs such as lactones [33,34], carbonates [35], disulfides [36], thioacetals [37,38] and olefins [39].

**Scheme 1.**
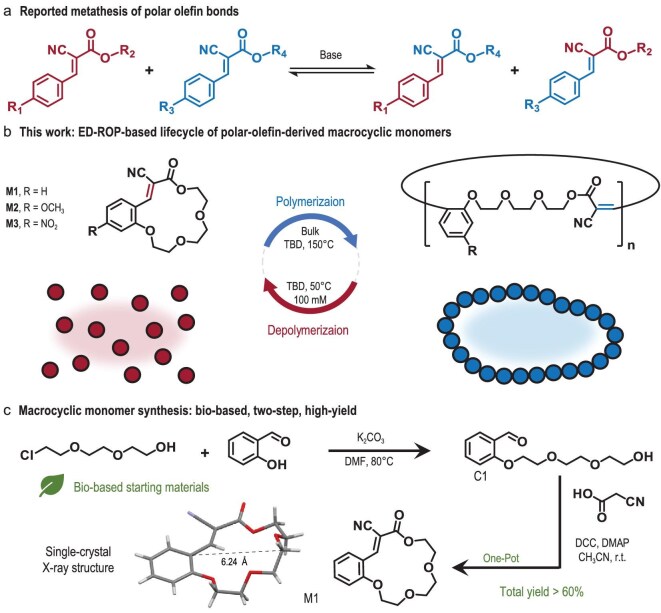
(a) Base-catalyzed dynamic covalent metathesis of polar olefin bonds [44]. (b) Chemical structures and schematic illustration of the present ED-ROP and closed-loop chemical recycling of macrocyclic monomers enabled by polar-olefin metathesis. (c) Synthetic route and single-crystal X-ray structure of the macrocycle M1.

Non-polar olefin bonds have been widely employed in the synthesis of degradable polymers via olefin metathesis polymerizations, driven by either enthalpic or entropic factors. These processes typically require Grubbs-type metal catalysts, which not only incur high costs but also necessitate tedious purification procedures to remove residual heavy metals, potentially limiting the applications of these materials [31,39]. In contrast, the DCC of Knoevenagel-type polar olefin bonds, involving conjugate addition with thiols [40], metathesis with imines [41,42], condensation with hydrazides [43], and self-metathesis (Scheme 1a) [44,45], exhibits remarkable dynamic behavior characterized by nucleophilic attack with low activation barriers, owing to the highly polarized electron density distribution within these bonds. Notably, these processes can proceed under metal-free catalytic conditions or even completely catalyst-free conditions [46,47]. This unique reactivity enables the easy synthesis of networked polymers under cost-effective and mild reaction environments, while simultaneously endowing the materials with versatile dynamic functionalities, including pluripotent mechanical properties [40], shape memory effects [42] and efficient mechanical recyclability [43].

This dynamic behavior motivates the reversible ROP-based lifecycle design of recyclable polymers featuring dynamic polar olefin bonds. Such materials are expected to exhibit remarkable reactivity, enabling either depolymerization back to monomers or post-polymerization modifications to form networked dynamic polymers, both processes reversible upon precise control of equilibrium. Nevertheless, despite these promising dynamic properties, such systems remain underexplored. Herein, we report the ED-ROP-based lifecycle of a novel bio-based macrocyclic monomer (M1) containing a dynamic covalent polar olefin bond. ED-ROP was successfully achieved via 1,5,7-triazabicyclo[4.4.0]dec-5-ene (TBD)-catalyzed metathesis of olefin bonds under melt *in situ* conditions (Scheme 1b). Structural characterization confirmed the formation of cyclic polymer topologies through monomer insertion and ring expansion. The dynamic polar olefin bonds facilitated efficient depolymerization under dilute conditions [50°C, 100 mM in chloroform (CHCl_3_)], allowing convenient monomer recovery and repolymerization following simple purification.

## RESULTS AND DISCUSSION

### Design, synthesis and characterization of monomers

Conventional petroleum-derived polymers face significant challenges in end-of-life management, contributing to substantial resource depletion and environmental contamination. To promote sustainable development, biomass-derived feedstocks represent a crucial alternative [48]. Accordingly, the present monomer was designed to adopt a synthetic route starting from bio-based salicylaldehyde and 2-[2-(2-chloroethoxy)ethoxy]ethanol, yielding intermediate C1 via Williamson etherification (Scheme 1c). Notably, salicylaldehyde is directly obtained from biomass [15], while the chloroethoxy precursor can be efficiently synthesized from bio-ethylene oxide through established high-yield procedures [49]. Meanwhile, incorporation of oligoethylene glycol chains with high flexibility is expected to effectively relieve strain within the macrocycle and increase conformational entropy upon polymerization. The incorporation of aromatic rings into the polymer backbone, a feature uncommon in closed-loop polymer design, is intended not only to improve synthetic accessibility by stabilizing aldehyde-containing precursors, but also to raise the glass-transition temperature (*T*_g_) and enhance mechanical properties. The intermediate C1 was subsequently converted into the macrocyclic monomer M1 through a one-pot esterification-to-Knoevenagel condensation tandem reaction, followed by straightforward purification involving filtration, washing and recrystallization, without requiring column chromatography. This synthetic approach affords M1 in a high overall yield exceeding 60%, and is readily scalable to quantities over 10 g. To demonstrate the generality of the present lifecycle design, we also synthesized analogous monomers M2 and M3, derived from 2-hydroxy-4-methoxybenzaldehyde and 2-hydroxy-4-nitrobenzaldehyde, respectively.

The monomer structures were characterized by ^1^H/^13^C NMR and high-resolution mass spectrometry (HRMS), with detailed data provided in the Supplementary data. NMR analysis confirmed the E-configuration of the macrocyclic olefin bonds in solution, with no detectable impurities. Single-crystal X-ray diffraction of M1 further revealed predominant retention of the E-configuration in the solid state. M1 features a cavity of 6.24 Å, providing a modest yet sufficient space around the polarized olefin bonds (Scheme 1c, Fig. S2) to accommodate nucleophiles for polar-olefin metathesis reactions.

### Entropy-driven ring-opening polymerization studies

**Figure 1.**
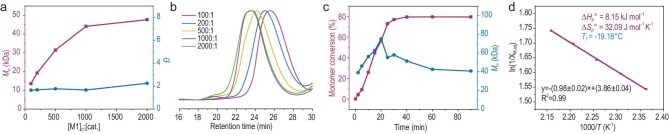
(a) Number-average *M*_n_ and *Đ* of Poly-M1 as a function of the [M1]:[TBD] ratio. (b) Overlaid GPC profiles of Poly-M1 at various [M1]:[TBD] ratios under bulk polymerization conditions at 150°C. (c) Plots of monomer conversion and *M*_n_ as a function of reaction time. (d) Van’t Hoff analysis of the polymerization of M1 under bulk conditions.

Wang *et al*. previously demonstrated alkaline-catalyzed metathesis of polar olefin bonds, establishing the foundation for ED-ROP of polar olefin-derived macrocycles (Scheme 1a) [44]. The proposed mechanism of this dynamic covalent process involves a nucleophilic attack by TBD on the polar olefin bond to form an intermediate anion. This intermediate subsequently attacks another polar olefin bond to generate a four-membered ring intermediate, which then undergoes ring-opening to yield the exchange products. In our initial catalyst screening, this non-toxic and highly active organic base was also identified as the optimal catalyst. Polymerizations were then conducted under bulk conditions at 150°C, above the melting point of M1 (132°C, Fig. S3), to maximize reaction efficiency. Notably, the environmental stability of TBD allowed polymerizations to proceed under convenient laboratory conditions without the need for rigorous anhydrous or oxygen-free protocols, thereby significantly simplifying the procedure. ^1^H NMR analysis showed that M1 conversion reached 79.5% within 1 h at a monomer-to-catalyst ratio ([M1]:[TBD]) of 100:1 at 150°C, yielding Poly-M1 with a number-average molar mass (*M*_n_) of 13.5 kDa and a dispersity index (*Đ*) of 1.65 (Fig. 1a and b; Table 1, entry 1), as determined by gel permeation chromatography (GPC). Systematic variation of the [M1]:[TBD] ratio from 200:1 to 2000:1 under identical conditions revealed a proportional increase in *M*_n_, reaching up to 47.7 kDa, while maintaining low dispersities (*Đ* < 1.8) below the 1000:1 ratio threshold. Increasing the polymerization temperature to 170°C only marginally improved conversion to 80.7%. The polymerization conducted in dimethylsulfoxide (DMSO) solution (10 M) required 5.5 h and resulted in a lower *M*_n_ of 19 kDa, likely due to reduced entropic driving forces compared to bulk conditions. Furthermore, the ED-ROP methodology proved generalizable to monomers M2 and M3, which polymerized at 170°C (*M*_n_ = 24.3 kDa, *Đ* = 1.47) and 190°C (*M*_n_ = 33.3 kDa, *Đ* = 1.91), respectively, demonstrating the versatility of this polymerization approach (Fig. S4).

**Table 1.** ED-ROP data for macrocyclic monomers catalyzed by TBD.

Entry	Monomer	[M]:[Catalyst]	Concentration^a^	Temperature (°C)	Time (h)	Conversion^b^ (%)	*M*_n,GPC_^c^ (kDa)	*Đ* ^c^
1	M1	100:1	Bulk	150	1	79.5	13.5	1.65
2	M1	200:1	Bulk	150	1	79.4	19.3	1.68
3	M1	500:1	Bulk	150	1	79.1	31.5	1.77
4	M1	1000:1	Bulk	150	1	79.6	44.1	1.67
5	M1	2000:1	Bulk	150	4	79.7	47.7	2.25
6	M1	1000:1	bulk	170	1	80.7	41.3	1.70
7	M1	1000:1	10 M in DMSO	150	5.5	77.3	19.0	1.60
8	M2	1000:1	Bulk	170	1	77.9	24.3	1.47
9	M3	1000:1	Bulk	190	1	81.5	33.3	1.91
10	ω-Pentadecalactone	1000:1	Bulk	190	4	0		

a Bulk indicates that the polymerization was carried out in solvent-free, *in situ* melting conditions. In the ‘10 M in DMSO’ entry, the concentration is expressed as *n_M1_*/*v_DMSO_*. ^b^Monomer conversion was determined by ^1^H NMR spectroscopy. ^c^Number-average *M*_n_ and *Đ* (*M*_w_/*M*_n_) were determined by GPC in THF relative to using polystyrene standards for calibration. Empty cells indicate no polymer formation.

Given that ester bonds represent another common class of base-catalyzed dynamic covalent bonds widely employed in ED-ROP [33], their potential contribution to the polymerization processes of M1–M3 warrants careful consideration. To investigate this, control experiments were conducted using a commercially available ω-pentadecalactone monomer (with a ring size similar to M1) that is known to undergo ED-ROP catalyzed by TBD. Under the tested conditions, no polymerization could be observed, even after 4 h at 190°C (Table 1, entry 10; Fig. S5), consistent with previous literature reports [50]. This can be attributed to the transesterification mechanism characteristic of lactone ROP, which proceeds via a nucleophilic addition–elimination pathway that necessitates free alcohol groups as essential reaction partners. In contrast, the metathesis polar olefin bonds occur only in the presence of a base catalyst and do not require free alcohol groups. Therefore, the reversible ED-ROP observed for M1–M3, which lack free alcohol groups to initiate transesterification, proceeds solely through the metathesis of polar olefin bonds.

The polymerization kinetics of M1 was then investigated by monitoring the evolution of molecular weight and monomer conversion as a function of time (Fig. 1c). Conversion increased steadily from 10% to approximately 80% over the course of 5 min to 1.5 h. Notably, the *M*_n_ exhibited non-monotonic behavior: an initial increase followed by a decline and eventual plateau (Figs S6 and S7). This kinetic profile distinguishes ED-ROP of polar olefin macrocycles from conventional macrocyclic lactone polymerizations [33]. The polymerization mechanism could be characterized by chain growth rather than living polymerization, attributed to competing pathways involving ring-opening, and cross- and ring-closing metathesis processes occurring between monomeric and polymeric olefin bonds. At the early stage, the high monomer concentration favors ring-opening metathesis, resulting in an increase in molecular weight. As conversion proceeds and polymer chain concentration rises, cross- and ring-closing metathesis pathways become predominant, leading to a reduction in molecular weight until the system reaches equilibrium. Exploiting these kinetic differences allows for strategic intervention to maximize *M*_n_ by quenching the reaction prior to equilibrium. For example, quenching at 20 min is expected to yield polymers with *M*_n_ approaching 80 kDa.

The equilibrium molar fraction (*X*_M,eq_) of M1 was then determined during bulk polymerization at various temperatures (150°C, 160°C, 170°C, 180°C and 190°C) with an [M1]:[TBD] ratio of 1000:1 (Fig. S8). Van’t Hoff analysis, plotting ln(1/*X*_M,eq_) versus 1000/T, afforded the standard thermodynamic parameters under bulk conditions: an enthalpy change Δ*H*_p_° of 8.15 kJ mol^−1^, and an entropy change Δ*S*_p_° of 32.09 J mol^−1^ K^−1^. Based on these values, *T*_f_ was calculated to be −19.18°C (Fig. 1d). The positive Δ*H*_p_° can be attributed to the intramolecular hydrogen bonding between the vinyl hydrogen and adjacent oxygen atoms. For monomers M2 and M3, the corresponding thermodynamic parameters were determined using the same method. Compared to M1, M2 exhibits a higher polymerization enthalpy (Δ*H*_p_° = 9.23 kJ mol^−1^) and a higher *T*_f_ of 5.79°C, whereas M3 shows a lower Δ*H*_p_° of 6.65 kJ mol^−1^ and a significantly reduced *T*_f_ of −43.28°C. This trend in enthalpy can be attributed to the modulation of push–pull conjugation by the substituents: the electron‑donating –OCH_3_ strengthens the push–pull interaction and thereby stabilizes the monomer, whereas the electron‑withdrawing –NO_2_ has the opposite effect. The significantly lower *T*_f_ of M3 can be attributed to the enhanced electron deficiency of the dynamic covalent olefin bond by –NO_2_, which remarkably reduces the activation energy and kinetic barrier for polymerization. This interpretation is also supported by the polymerization kinetics of the three monomers (Fig. S12): at 190°C, M3 polymerizes at the highest rate, followed by M1, while M2 exhibits the slowest polymerization rate.

**Figure 2.**
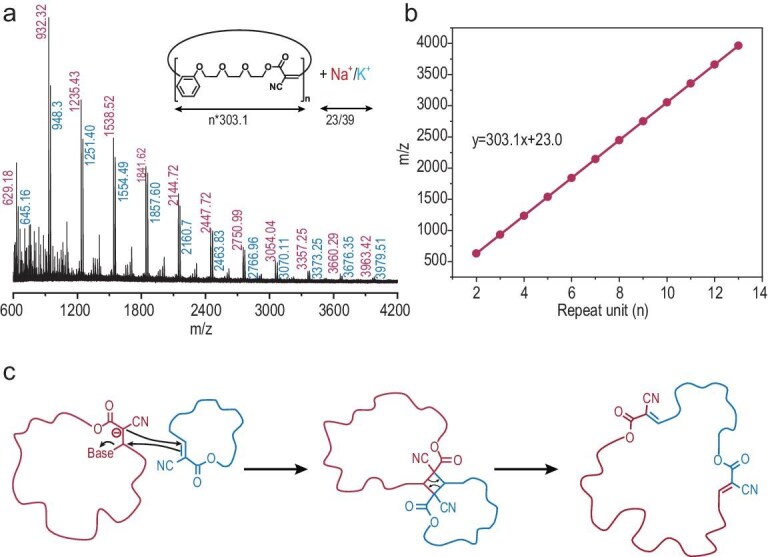
(a) MALDI-TOF MS spectrum of Poly-M1 in the low molecular weight range. (b) Plot of *m*/*z* values of Poly-M1 versus the theoretical number of repeat units. (c) Proposed mechanism of insertion and ring-expansion polymerization for macrocycles containing polar olefin bonds.

The Poly-M1 sample prepared at an [M1]:[TBD] ratio of 1000:1 was then analyzed by matrix-assisted laser desorption/ionization time-of-flight mass spectroscopy (MALDI-TOF MS), which successfully detected mass spectral signals corresponding to the low-molecular-weight fraction of the polymer. As shown in Fig. 2a, the mass spectrum exhibits two well-defined series of molecular ion peaks, with consecutive peaks in each series separated by 303.1 Da, in line with the theoretical molar mass of the monomer M1. Furthermore, a linear correlation between the *m*/*z* values and the degree of polymerization (number of repeating units) was established (Fig. 2b and Fig. S9). Both fitted lines exhibit identical slopes of 303.1 Da per repeat unit, with intercepts at 23 and 39 Da, corresponding to sodium and potassium cation adducts, respectively. These analytical results provide conclusive evidence for the formation of polymers bearing cyclic topologies. Considering the absence of external initiators and end groups, alongside the comprehensive kinetic data and MALDI-TOF MS characterization, we propose that the polymerization of polar-olefin-containing macrocycles proceeds predominantly via an insertion and ring-expansion mechanism (Fig. 2c), ultimately equilibrated to yield polymers with well-defined cyclic architectures.

### Polymer characterization

**Figure 3.**
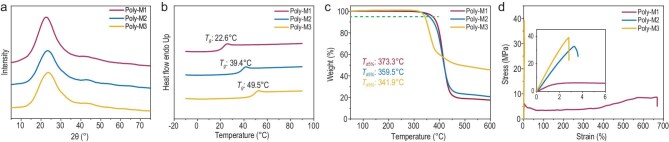
(a) XRD, (b) DSC, (c) TGA and (d) stress–strain curves of Poly-M1 to Poly-M3 (Poly-M1: *M*_n_ = 44.1 kDa, *Đ* = 1.67; Poly-M2: *M*_n_ = 24.3 kDa, *Đ* = 1.47; Poly-M3: *M*_n_ = 33.3 kDa, *Đ* = 1.91).

The three polymers (Poly-M1 to Poly-M3; see entries 4, 8 and 9 in Table 1 for synthetic conditions) were characterized using multiple analytical techniques. Both ^1^H and ^13^C NMR spectra displayed single sets of signals, confirming the retention of the E-configuration in the polar olefin bonds throughout polymerization (Figs S13–S18). X-ray diffraction (XRD) patterns exhibited broad peaks characteristic of amorphous materials (Fig. 3a). Differential scanning calorimetry (DSC) analysis revealed distinct *T*_g_ values at 22.6°C (Poly-M1), 39.4°C (Poly-M2) and 49.5°C (Poly-M3), with no observable melting endotherms. The variation in *T*_g_ primarily arises from structural differences in side-chain substituents, where the introduction of –OCH_3_ and –NO_2_ groups significantly enhances *T*_g_ values (Fig. 3b). Thermal stability was assessed by thermogravimetry analysis (TGA). All three polymers exhibited excellent thermal stability, with 5% weight loss temperatures (*T*_d5%_) values of 373.3°C (Poly-M1), 359.5°C (Poly-M2) and 341.9°C (Poly-M3) (Fig. 3c). Differential thermogravimetry (DTG) analysis revealed maximum decomposition temperatures (*T*_max_) at 414.1°C, 412.2°C and 360.5°C, respectively (Fig. S19), underscoring the exceptional thermal stability of ED-ROP-derived polymers.

The polymers were then compression-molded into films and subjected to uniaxial tensile testing to evaluate their mechanical properties (Fig. 3d). The introduction of substituents on the benzene rings significantly influenced the mechanical performance of the produced polymers. Both Poly-M2 (tensile strength (*σ*) = 31.39 ± 2.79 MPa; Young’s modulus (*E*) = 984.2 ± 141.5 MPa) and Poly-M3 (*σ* = 39.09 ± 2.66 MPa; *E* = 1780.2 ± 205.9 MPa) exhibited notably higher tensile strength and modulus compared to Poly-M1 (*σ* = 7.92 ± 0.94 MPa; *E* = 308.9 ± 61.0 MPa). However, this mechanical reinforcement was accompanied by significant embrittlement, as evidenced by a drastic reduction in elongation at break (3.4% ± 0.2% for Poly-M2 and 3.0% ± 0.5% for Poly-M3, versus 621.1% ± 86.2% for Poly-M1).

### Closed-loop chemical recycling studies

The above calculated thermodynamic parameters indicate that the polymerization driving force of M1 under bulk melt polymerization conditions primarily arises from an increase in conformational entropy. This also suggests that Poly-M1 could potentially undergo depolymerization and regenerate monomers under dilute-solution conditions, where the conformational entropy contribution is minimized and the translational entropy (positive in depolymerization) becomes a significant driving force, i.e. Δ*S* > 0 during depolymerization. Accordingly, Poly-M1 was dissolved in CHCl_3_ at a concentration of 100 mM (for polar olefin bonds), with TBD as the catalyst (10 mol% relative to olefin bonds) to initiate depolymerization at 50°C. The depolymerization process was monitored by time-dependent ¹H NMR and GPC.

**Figure 4.**
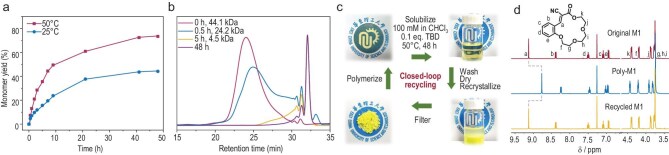
(a) Monomer yield as a function of depolymerization time for Poly-M1 in CDCl_3_ (100 mM, 10 mol% TBD) at 50 and 25°C. (b) GPC traces of Poly-M1 at various depolymerization times. (c) The closed-loop chemical recycling process of Poly-M1 back to monomer. (d) Comparative ¹H NMR spectra of the original M1, Poly-M1, and recycled M1.

The vinyl proton signals in ^1^H NMR allow clear differentiation between monomeric and polymeric species. For M1, due to significant electron shielding of the vinyl proton H_a_ located closely between two oxygen atoms (2.197 and 2.257 Å as determined by the single crystal structure of M1), the vinyl peak appears downfield at 9.13 ppm (Fig. 4d). In contrast, oligomeric and polymeric macrocycles adopt more extended conformations that reduce this shielding effect, resulting in an upfield shift of vinyl protons to the 8.70–8.85 ppm region. Accordingly, the formation of monomer M1 was detected at the early stage of depolymerization (0.5 h), with its signal intensity increasing over time, while polymer signals gradually diminished (Fig. S20). Additionally, new peak clusters appeared during depolymerization, likely corresponding to macrocyclic oligomers of varying sizes formed as unavoidable thermodynamic equilibration products, supported by higher retention time peaks in the GPC traces (Fig. 4b). Kinetic curves (Fig. 4a) showed that after 21 h, polymer signals were nearly undetectable, yet monomer yield reached 60%. Extending depolymerization to 48 h further increased monomer yield to 73%, almost reaching equilibrium, with residual macrocyclic oligomers remaining. Lowering the temperature to 25°C significantly slowed the depolymerization rate, resulting in a reduced monomer yield of 44% after 48 h (Fig. S21), consistent with the thermodynamic expectation that a positive Δ*S* leads to an increased Δ*G* at lower temperatures. Moreover, replacing TBD with triethylamine (TEA) resulted in monomer yields below 5% after 48 h (Fig. S22), likely due to the weaker basicity of TEA, which is insufficient to effectively lower the energy barrier and thus markedly slows depolymerization kinetics.

The chemical recycling procedure was then attempted and optimized (Fig. 4c); 303 mg of Poly-M1 was dissolved in 10 mL of CHCl_3_ and reacted at 50°C for 48 h, followed by washing, drying and recrystallization to afford a light-yellow powder in 63% yield. The ^1^H NMR spectrum of this recovered material (Fig. 4d) clearly confirmed the presence of pure monomer M1 compared to the original material, as evidenced by the characteristic vinyl peak at 9.13 ppm. The moderate monomer recovery is attributed to the residual oligomer formation at equilibrium. Inspired by the work of Kariyawasam *et al.* [37] and Nieboer *et al.* [51], who demonstrated that mixtures of different-sized macrocycles containing thioacetal or lactones can still undergo ED-ROP, we attempted to directly repolymerize the crude depolymerization product of Poly-M1. After washing with water and drying, without the need for complex purification, we obtained a mixture composed mainly of M1 and its dimer (Fig. S23). This mixture was successfully repolymerized using TBD as a catalyst. Successful chain extension was confirmed by ^1^H NMR and GPC (Figs S24 and S25), supporting the feasibility of a separation-free recycling process.

## CONCLUSION

In this study, we established a novel closed-loop recycling system for biobased macrocyclic monomers by harnessing dynamic polar olefin bonds. Starting from salicylaldehyde and ethylene oxide derivatives, the bio-based monomer M1 and its derivatives were synthesized via an efficient route with an overall yield exceeding 60%. Utilizing TBD as the catalyst, these monomers underwent ED-ROP under melt conditions, yielding high-molecular-weight cyclic polymers (*M*_n_ up to 47.7 kDa). Kinetic analysis revealed a chain-growth polymerization mechanism governed by the competitive interplay among ring-opening, and cross- and ring-closing metathesis pathways. Thermodynamic studies confirmed the entropy-driven nature of the process (Δ*H*_p_° = 8.15 kJ mol^−1^, Δ*S*_p_° = 32.09 J mol^−1^ K^−1^, *T*_f_ = −19.18°C). Leveraging the reversibility of polar-olefin metathesis, efficient depolymerization was achieved under dilute conditions (50°C, CHCl_3_, 100 mM) with a monomer regeneration efficiency of 63%. This approach further expands the DCC of polar olefin bonds featuring reduced activation barriers. Future efforts may focus on modifying ring structures to enable catalyst-free reaction conditions, as well as harnessing these dynamic linkers within the framework of constitutional dynamic chemistry [52], potentially accelerating the development of adaptive polymers with multiple dynamic functions.

## Supplementary Material

nwaf484_Supplemental_Files
