# Supplementary material for: Lifecycle of dynamic covalent polar-olefin macrocycles via entropy-driven ring-opening polymerization and closed-loop chemical recycling
Source: Natl Sci Rev. 2025 Nov 6;12(12):nwaf484. doi: 10.1093/nsr/nwaf484 (PMC12715864; doi:10.1093/nsr/nwaf484)
Supplement: nwaf484_Supplemental_Files [file nwaf484_supplemental_files.zip › 1508-SI.pdf]

Supporting Information

Lifecycle of Dynamic Covalent Polar-Olefin Macrocycles via Entropy-Driven Ring-Opening Polymerization and Closed-Loop Chemical Recycling

Pengyun Li[1], Chong Li[1], Mengying Lei[1], Ruirui Gu[1]*, He Tian[1], Da-Hui Qu[1]*

[1]Key Laboratory for Advanced Materials and Joint International Research Laboratory of Precision Chemistry and Molecular Engineering, Feringa Nobel Prize Scientist Joint Research Center, Frontiers Science Center for Materiobiology and Dynamic Chemistry, Institute of Fine Chemicals, School of Chemistry and Molecular Engineering, East China University of Science and Technology, 200237 Shanghai, P. R. China

E-mails of corresponding authors: dahui_qu@ecust.edu.cn, guruirui@ecust.edu.cn

Contents

1.Experimental details **3**

1.1 Materials 3

1.2 General characterization methods 3

1.3 Synthesis of monomers 4

1.4 General Polymerization Procedure of M 1-3 6

1.5 Thermodynamics Experiments of M1 7

1.6 Depolymerization of Poly-M1 8

2.Supporting Figures and Tables **10**

Appendix **25**

1.Experimental details

1.1 Materials

Salicylaldehyde，2-(2-(2-chloroethoxy)ethoxy)ethanol, 2-hydroxy-4-methoxy-benz-aldehyde, 2-hydroxy-4-nitrobenzaldehyde, cyanoacetic acid, 4-dimethylaminopyridine (DMAP), N, N'-dicyclohexylcarbodiimide (DCC), 1,5,7-triazabicyclo [4.4.0] dec -5-ene (TBD), triethylamine (TEA), cyclopentadecanolide, dimethylformamide, ethyl acetate, acetonitrile, dichloromethane, methanol, dimethyl sulfoxide, potassium carbonate, sodium hydroxide. All the reagents were purchased commercially and were used without further purification if it is not noted.

1.2 General characterization methods

Nuclear magnetic resonance (NMR) spectra were recorded on a Bruker AV-III-400 or a Bruker 600 spectrometer at ambient temperature (298 K). Chemical shifts for ^{1}H and ^{13}C NMR spectra were referenced to the internal solvent resonances and were reported as parts per million (ppm) relative to tetramethylsilane.

High-resolution mass spectra (HRMS) were recorded on a Waters Xevo G2 Tof mass spectrometer using an electrospray ionization source.

X-ray diffraction (XRD) date of polymer films was conducted on a Rotating Anode X-ray Diffractometer (18KW/D/max2550VB/PC) using Cu Kα radiation (λ = 1.5406 Å) at a scan speed of 5 °·min^{-1} from 5 to 75 °.

The molecular weight test (polymer weight-average molar mass (Mw), polymer number-average molar mass (M_n) and dispersity ($Đ = M_w/M_n$)) of the polymer were performed via **Gel Permeation Chromatography (GPC)**. GPC equipment consists of a Waters HPLC system equipped with three Waters Styragel columns (pore sizes are 104, 103 and 500 Å, respectively) and coupled with a Waters 2414 differential detector. The analysis was performed at 35 ℃ with THF as the eluent at a flow rate of 1.0 mL/min. Data analysis was acquired using SPA 3.30 software calibrated with low dispersity polystyrene standards.

Thermo gravimetric Analysis (TGA) was carried out with a Mettler Toledo TGA/

SDTA851. Polymer samples were heated under N_2 atmosphere from ambient temperature to 600 °C at a heating rate of 10 ℃ min^{-1}.

Differential scanning calorimetry (DSC) measurements were carried out with a DSC 300 Caliris (Netzsch instruments). The melting point of the monomer was determined under a nitrogen atmosphere. A sample (approximately 5 mg) was heated at a rate of 10 ℃/min from 20 ℃ to 180 ℃, and the melting point was determined by the position of the endothermic peak. Furthermore, the polymer samples (around 5-10 mg) were heated from 25 to 100 ℃ at a heating rate of 10 ℃ min^{-1}, kept at 100 ℃ for 3 min to eliminate the thermal history, cooled to -20 ℃ at a cooling rate of 20 ℃ min^{-1}, and then heated to 100 ℃ at a heating rate of 10 ℃ min^{-1}. The glass transition temperature (T_g) was measured by taking the midpoint of the reversible endotherm of the second heating curve for each sample.

Uniaxial tensile tests were performed on a SUNs UTM4104 tensile instrument. The dimensions of the samples were approximately 50 mm (l) × 5 mm (w) × 0.2 mm (t). The strain rate was 5 mm min^{-1}. The tests of each sample were repeated 5 times at least.

1.3 Synthesis of monomers

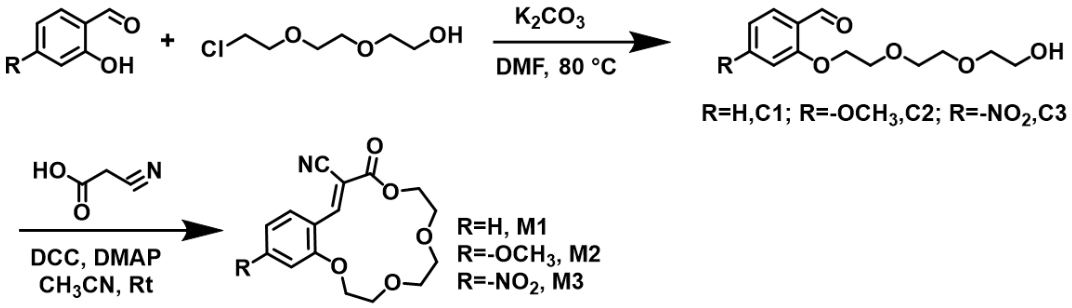

Scheme S1. The synthetic route of macrocycle monomers containing polar olefin bonds.

To a 250 ml round-bottom flask with 60 ml DMF, 3.36g 2-(2-(2-chloroethoxy) ethoxy) ethanol (0.02 mol 1 eq), 2.68 g salicylaldehyde (0.022 mol 1.1 eq) and 4.14 g K_2CO_3 (0.03 mol, 1.5 eq) were added and allowed to stir overnight at 80 ℃. After cooling to room temperature, the suspension was filtered, and the solid was washed with DMF (30 mL). Then EtOAc was added, and the organic phase was washed with 1 M NaOH solution followed by deionized water. The organic layer was then dried over Na_2SO_4

and filtered, 4.1g of light-yellow transparent liquid compound C1was obtained after concentrated under reduced pressure (yield: 78%). Using the same method, compounds C2 (yield: 81%) and C3 (yield: 88.4%) were obtained starting from 2-hydroxy-4-methoxybenzaldehyde and 2-hydroxy-4-nitrobenzaldehyde respectively.

C1: ^{1}H NMR (400 MHz, $CDCl_3$) δ 10.52 (s, 1H), 7.83 (dd, J = 7.7, 1.8 Hz, 1H), 7.53 (ddd, J = 9.0, 7.4, 1.8 Hz, 1H), 7.04 (t, J = 7.5 Hz, 1H), 6.99 (d, J = 8.4 Hz, 1H), 4.29 – 4.24 (m, 2H), 3.93 – 3.89 (m, 2H), 3.74 – 3.67 (m, 6H), 3.62 – 3.59 (m, 2H). ^{13}C NMR (151 MHz, $CDCl_3$) δ 190.11, 161.20, 135.95, 128.32, 125.19, 121.10, 113.07, 77.33, 77.12, 76.91, 72.60, 70.98, 70.39, 69.45, 68.31, 61.71. HRMS (ESI+): m/z calcd. for $[M+Na]^+$: 277.1042, found:277.1041.

C2: ^{1}H NMR (400 MHz, $CDCl_3$)10.32 (s, 1H), 7.80 (d, *J* = 8.7 Hz, 1H), 6.55 (dd, *J* = 8.7, 1.7 Hz, 1H), 6.45 (d, *J* = 2.2 Hz, 1H), 4.24 – 4.18 (m, 2H), 3.92 – 3.87 (m, 2H), 3.85 (s, 3H), 3.75 – 3.66 (m, 6H), 3.61 – 3.57 (m, 2H). 13C NMR (151 MHz, CDCl3) δ 188.57, 166.11, 162.98, 130.30, 119.23, 106.45, 99.05, 77.36, 77.15, 76.94, 72.61, 70.98, 70.38, 69.37, 68.31, 61.67, 55.64. HRMS (ESI+): m/z calcd. for $[M+Na]^+$: 307.1158, found:304.1145.

C3:^{1}H NMR (400 MHz, $CDCl_3$) δ 10.56 (s, 1H), 7.99 (d, *J* = 8.4 Hz, 1H), 7.93 – 7.85 (m, 2H), 4.42 – 4.36 (m, 2H), 3.99 – 3.94 (m, 2H), 3.77 – 3.71 (m, 6H), 3.61 (dd, *J* = 5.3, 3.8 Hz, 2H). 13C NMR (151 MHz, CDCl3) δ 188.60, 161.18, 152.08, 129.42, 128.91, 115.84, 108.61, 77.32, 77.11, 76.89, 71.01, 69.24, 69.14, 61.73, 61.69, 42.73. HRMS (ESI+): m/z calcd. for $[M+Na]^+$: 322.0903, found:322.0892.1145.

To a 250 mL round-bottom flask containing 100 mL of acetonitrile, 2.54 g of compound C1 (0.01 mol, 1 eq) and 1.02 g of cyanohexanoic acid (0.012 mol, 1.2 eq) were added and stirred until completely dissolved. The reaction flask was placed in an ice-water bath to cool, and then 2.47 g of 1,3-Dicyclohexylcarbodiimide (DCC) and 12 mg of 4-dimethylaminopyridine (DMAP) were added in batches. The mixture was then stirred at room temperature overnight. After the reaction was completed, the insoluble substances were filtered out, and the resulting filtrate was concentrated under reduced pressure. The concentrated solution was dissolved in ethyl acetate and

washed with deionized water five times. The organic layer was dried over anhydrous sodium sulfate, filtered, and concentrated to obtain the crude product. Finally, the crude product was recrystallized from methanol to obtain monomer M1, with a yield of 81%. Monomers M2 and M3 are obtained from C2 and C3 using the same method, with yields of 83% and 78%, respectively.

M1: ^{1}H NMR (400 MHz, $CDCl_3$) δ 9.15 (s, 1H), 8.41 (dd, J = 8.0, 1.6 Hz, 1H), 7.52 (ddd, J = 8.8, 7.4, 1.6 Hz, 1H), 7.11 (t, J = 7.6 Hz, 1H), 6.97 (d, J = 8.3 Hz, 1H), 4.44 – 4.40 (m, 2H), 4.23 – 4.20 (m, 2H), 3.91 – 3.88 (m, 2H), 3.81 – 3.77 (m, 6H).^{13}C NMR (151 MHz, CDCl3) δ 162.77, 158.71, 149.47, 135.14, 128.96, 121.69, 121.47, 115.50, 112.95, 101.44, 77.28, 77.07, 76.86, 70.03, 70.01, 69.09, 68.29, 68.22, 64.75. HRMS (ESI+): m/z calcd for $[M+H]^+$: 304.1185, found:304.1170.

M2: ^{1}H NMR (400 MHz, $CDCl_3$) δ 9.05 (s, 1H), 8.42 (d, J = 8.9 Hz, 1H), 6.62 (dd, J = 8.9, 2.4 Hz, 1H), 6.42 (d, J = 2.4 Hz, 1H), 4.39 – 4.34 (m, 2H), 4.19 – 4.14 (m, 2H), 3.88 (s, 5H), 3.78 – 3.74 (m, 6H).^{13}C NMR (151 MHz, $CDCl_3$) δ 165.74, 163.36, 160.73, 148.73, 130.76, 116.33, 114.67, 107.02, 99.26, 97.58, 77.29, 77.07, 76.86, 69.99, 69.89, 68.94, 68.37, 68.01, 64.48, 55.75. HRMS (ESI+): m/z calcd for $[M+H]^+$: 334.1291, found:334.1274.

M3: ^{1}H NMR (400 MHz, $CDCl_3$) δ 9.04 (s, 1H), 8.49 (d, J = 8.7 Hz, 1H), 7.95 (dd, J = 8.6, 2.2 Hz, 1H), 7.78 (d, J = 2.2 Hz, 1H), 4.45 – 4.40 (m, 2H), 4.33 – 4.29 (m, 2H), 3.94 – 3.90 (m, 2H), 3.76 (dtd, J = 11.6, 5.9, 5.1, 3.6 Hz, 6H).^{13}C NMR (151 MHz, $CDCl_3$) δ 161.67, 158.57, 151.07, 147.02, 129.56, 127.01, 116.44, 114.52, 107.59, 105.79, 77.25, 77.04, 76.83, 70.14, 70.00, 68.78, 68.59, 68.17, 65.11. HRMS (ESI+): m/z calcd for $[M+Na]^+$: 371.0855, found:371.0841.

1.4 General Polymerization Procedure of M 1-3

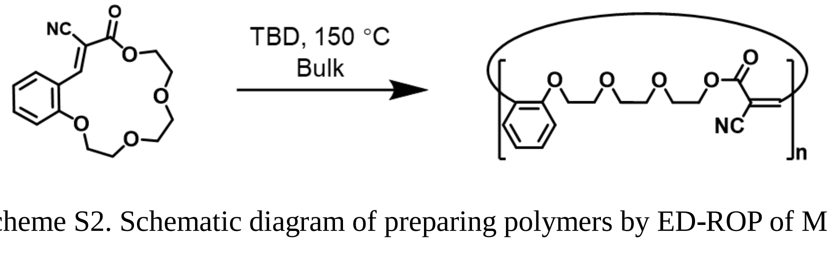

Scheme S2. Schematic diagram of preparing polymers by ED-ROP of M1.

The polymerization reactions in this work were carried out under conventional conditions

without the need for strict anhydrous and anoxic operations. After preliminary condition screening, the representative polymerization experimental steps are as follows: 151.6 mg of monomer M1 was placed in a 2 mL glass bottle equipped with a magnetic stirrer. Using a micropipette, 50 μL of CH_2Cl_2 solution containing TBD (with a concentration of 100 mM) was added to the bottle to achieve a monomer-to-catalyst molar ratio of 100:1. Subsequently, the reaction bottle was transferred to a 50 °C vacuum drying box, and the solvent CH_2Cl_2 was removed. After drying for 3 hours, the reaction bottle was taken out, sealed with a cap, and reacted at 150 ℃ for 1 hour. After the reaction, the reaction system was cooled to room temperature, and the obtained crude product was dissolved in 1 mL of CH_2Cl_2 (with 5 μL of acetic acid added beforehand). Then, the solution was dropped into 3 mL of ice methanol to precipitate the polymer. This dissolution-sedimentation purification cycle was repeated three times. Finally, the precipitate was vacuum-dried to obtain pure polymer (105.4 mg, yield 69.5%, calculated based on the monomer M1 input amount). For polymerization systems with different monomer/catalyst molar ratios (Table S1), the ratio was adjusted by changing the volume of the TBD/CH_2Cl_2 solution added (while keeping the TBD concentration at 100 mM). In addition, polymerization experiments in DMSO were conducted, with the difference being the addition of 100 μL of DMSO and the extension of the polymerization time to 5 hours.

Table S1. The formulations of the polymerization processes for different groups.

Entry	[M]:[Cat.]	Temperature (℃)	Monomer		TBD aq. (DCM,0.1 M) (μL)	Time (h)	Solvent
			Type	Mass (mg)			
1	100:1	150	M1	151.6	50	1	-
2	200:1	150	M1	151.6	25	1	-
3	500:1	150	M1	151.6	10	1	-
4	1000:1	150	M1	151.6	5	1	-
5	2000:1	150	M1	151.6	2.5	4	-
6	1000:1	150	M1	151.6	5	5.5	DMSO
7	1000:1	170	M2	166.8	5	1	-
8	1000:1	190	M3	174.1	5	1	-

1.5 Thermodynamics Experiments of M1

The polymerization thermodynamic experiment of M1 was conducted under normal conditions and with 0.1 mole% of TBD. The specific experimental steps are as follows: 151.6 mg of M1 and 5 μL of CH_2Cl_2 containing of TBD (100 mM) were added separately to 5 dry

glass vials with magnetic stir bars. Then, the reaction vials were transferred to a vacuum drying box at 50 °C to remove the CH_2Cl_2. After 3 hours of drying, the reaction vials were taken out, sealed with a cap, and reacted for 1 hour at 150, 160, 170, 180, and 190 ℃. After the reaction, a small amount of the mixture was dissolved in 600 microliters of $CDCl_3$ (with 2μL of acetic acid added beforehand for quenching) for ^1H NMR qualitative analysis. The monomer conversion rate was determined by integrating the characteristic signals of the polymer and monomer, and the monomer equilibrium molar fraction $X_{M,eq}$ under the bulk polymerization conditions was further determined. The data were then plotted as $ln\left(\frac{1}{X_{M,eq}}\right)$vs $\frac{1000}{T}$. Then, the equation $ln\left(\frac{1}{X_{M,eq}}\right) = -\frac{\Delta H_x^o}{RT} + \frac{\Delta S_x^o}{R}$ was applied for linear fitting to obtain the thermodynamic parameters.

1.6 Depolymerization of Poly-M1

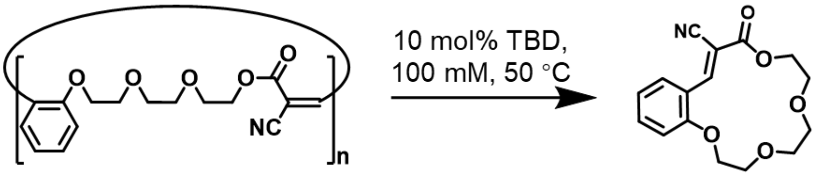

Scheme S3. A schematic diagram showing the closed-loop depolymerization of Poly-M1 into monomer M1.

Decomposition kinetics: Taking Poly-M1 as an example, kinetic experiments were conducted at 25 and 50 ℃ to systematically study the decomposition behavior of polymers containing polar olefin bonds. First, 30.3 mg of purified Poly-M1 and 1.39 mg of TBD were added to a 2 μL vial, followed by the addition of 1 ml of $CDCl_3$. The concentrations of polar olefin bonds and TBD were controlled at 100 and 10 mM, respectively. Then, it was placed in a water bath at 50°C and heated. After different reaction times, 50 μL of the mixed solution was taken and injected into 500 μL of $CDCl_3$ containing 1 μL of acetic acid (for quenching TBD). Subsequently, ^1H NMR spectroscopy was performed, and the monomer yield was determined by integrating the characteristic proton signal peaks, and the kinetic curves were plotted. In addition, a decomposition kinetics experiment at 25 ℃ was also conducted using the same method.

Poly-M1 depolymerization and monomer recovery experiment: 303 mg of purified

Poly-M1 and 13.9 mg of TBD were added to a 25 ml bottle, followed by the addition of 10 ml of $CHCl_3$. The mixture was heated at 50 ℃ for 48 hours. After the reaction was completed and the mixture cooled to room temperature, 20 μL of acetic acid was added to neutralize TBD. Then, the mixture was washed three times with deionized water, dried and concentrated to obtain the depolymerization crude product. Subsequently, the product was purified by recrystallization in methanol.

2.Supporting Figures and Tables

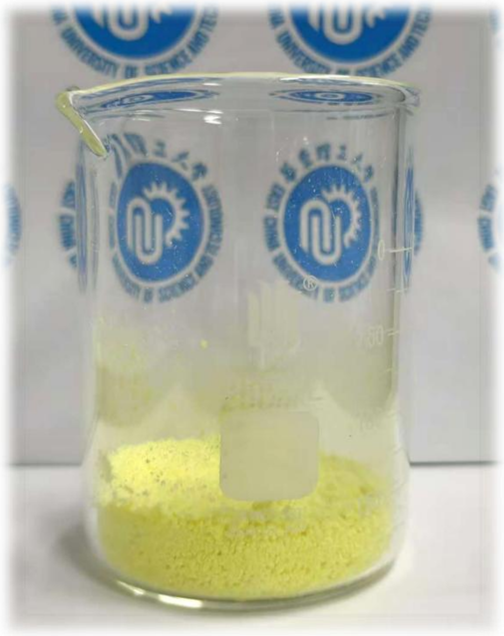

Figure S1. Obtain more than 10 g of M1 monomer in a single production batch.

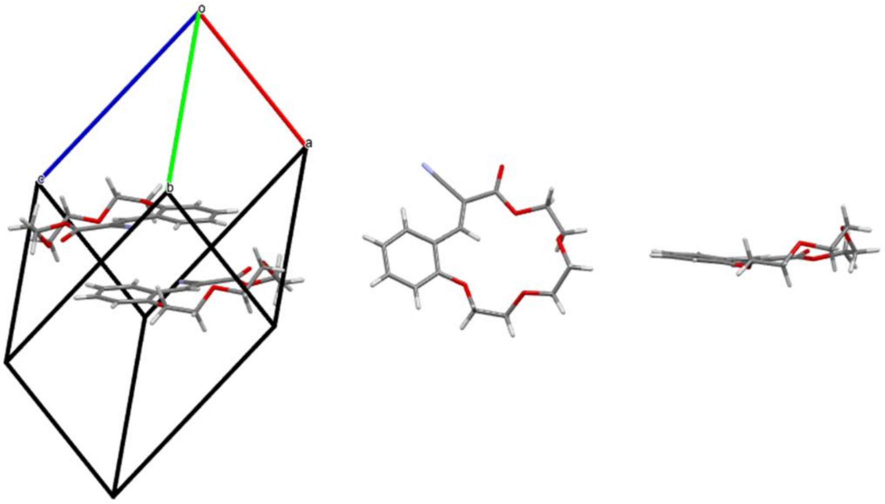

Figure S2. The single-crystal X-ray structure of M1 from different perspectives.

Table S2. Crystal data and structure refinement for M1

$C_{16}H_{17}NO_5$	*Z* = 2
M_r = 303.30	*F*(000) = 320
Triclinic, $P\bar{1}$	D_x = 1.325 Mg m^{-3}
a = 8.0682 (5) Å	Cu *K*a radiation, l = 1.54178 Å
b = 9.7274 (6) Å	Cell parameters from 6034 reflections
c = 11.5891 (7) Å	q = 4.2–68.5 °
a = 65.222 (3) °	m = 0.83 mm^{-1}
b = 76.269 (3) °	*T* = 200 K
g = 67.585 (3) °	Block, yellow
V = 760.15 (8) $Å^3$	0.05 × 0.04 × 0.01 mm

Table S3. Optimized structure of M1

O	10.834844	5.482273	3.097933
O	8.911498	9.011606	4.307701
O	8.886049	8.906292	1.515010
O	9.830957	6.298452	0.624733
O	10.957056	3.513725	4.140968
C	8.300439	8.666144	5.459698
C	8.616043	7.396504	5.958904
C	10.664296	4.668082	4.140109
C	8.368322	10.066574	3.504018
H	8.448161	10.935449	3.971845
H	7.412404	9.897660	3.309656
C	9.365933	6.547521	5.108367
H	9.484497	6.892303	4.231113
C	9.160171	10.076974	2.249251
H	8.924909	10.873625	1.710650
H	10.125927	10.120839	2.462367
C	9.942912	5.347402	5.301944

C	11.296596	4.912774	1.858211
H	10.732791	4.140815	1.600651
H	12.233518	4.605289	1.946230
C	8.034528	7.022038	7.193095
H	8.217163	6.165918	7.562143
C	9.969598	4.619765	6.523184
C	7.425700	9.512074	6.144015
H	7.190325	10.355066	5.774600
N	9.983439	4.026830	7.474962
C	11.196727	5.994884	0.837462
H	11.676234	6.801458	1.153076
H	11.612263	5.697846	-0.010613
C	6.910193	9.122326	7.346334
H	6.341996	9.713583	7.825979
C	7.197953	7.901605	7.866515
H	6.822409	7.646469	8.701001
C	9.795342	8.752750	0.439622
H	10.725380	8.804402	0.774986
H	9.662100	9.478562	-0.220325
C	9.560420	7.419949	-0.212173
H	8.616713	7.374040	-0.507818
H	10.130238	7.357424	-1.019335

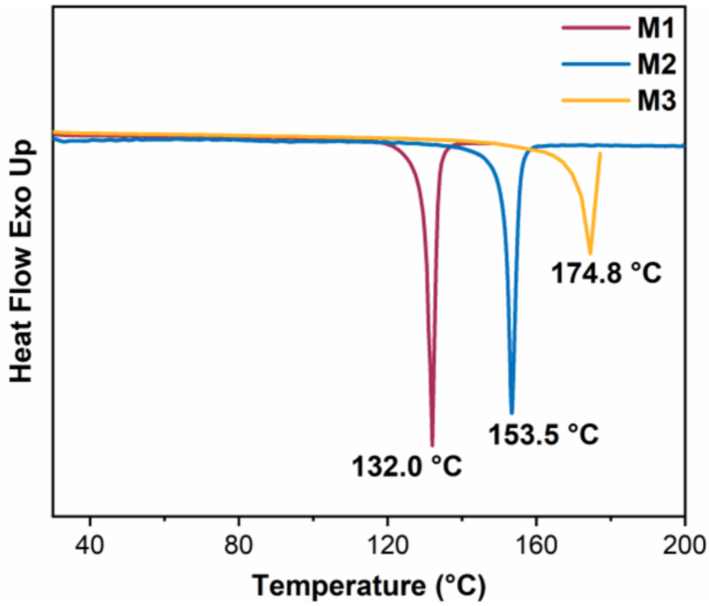

Figure S3. The melting points of M1 to M3 were measured by DSC.

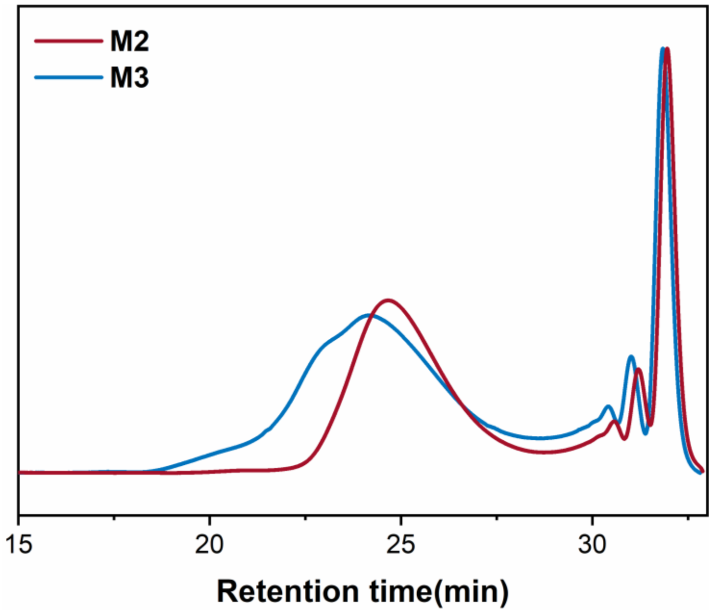

Figure S4. the GPC traces of M2 and M3 after reacting for 1 hour at 170 ℃ and 190 ℃ respectively within a [Monomer]:[TBD] ratio of 1000:1.

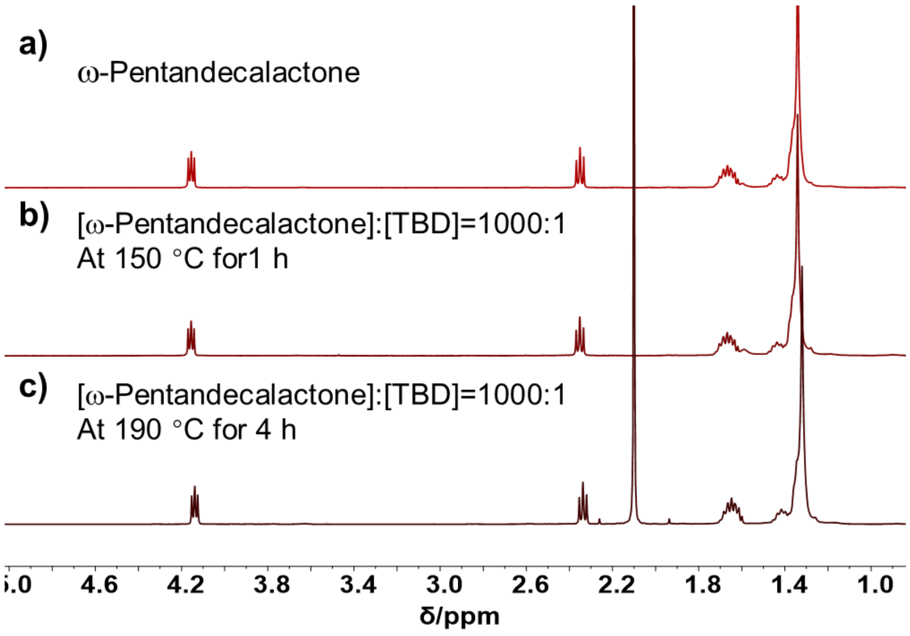

Figure S5. H NMR spectra of ω-pentandecalactone, the mixture ([ω-pentandecalac-tone]: [TBD] = 1000:1) after reaction b) at 150 ℃ for 1 h and c) at 190 ℃ for 4 h.

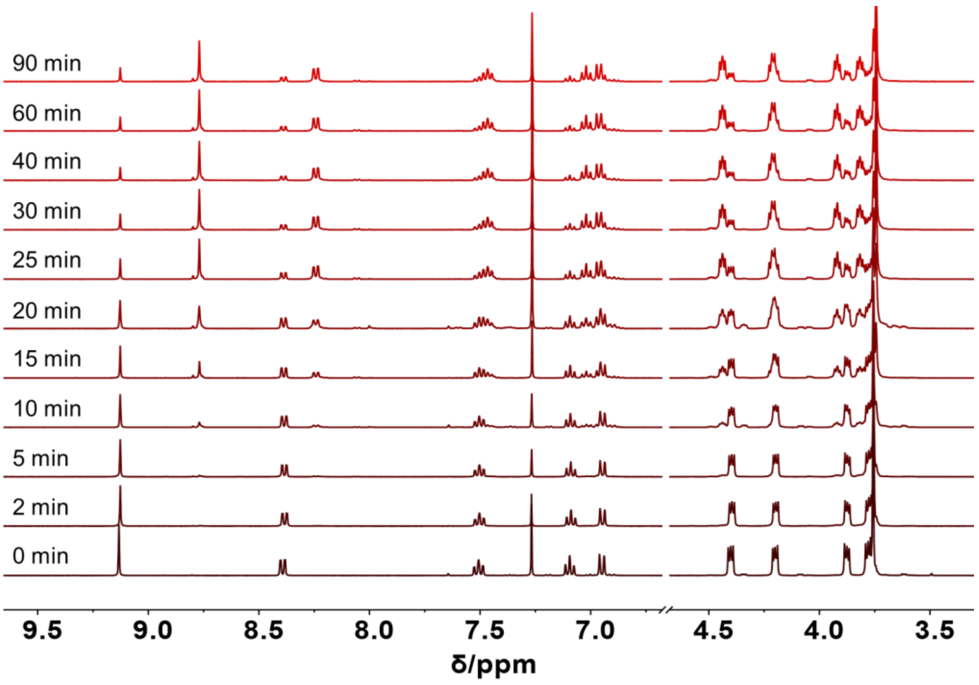

Figure S6. ^{1}H NMR spectra at different reaction times within a [M1]:[TBD] ratio of 1000:1 at 150 ℃.

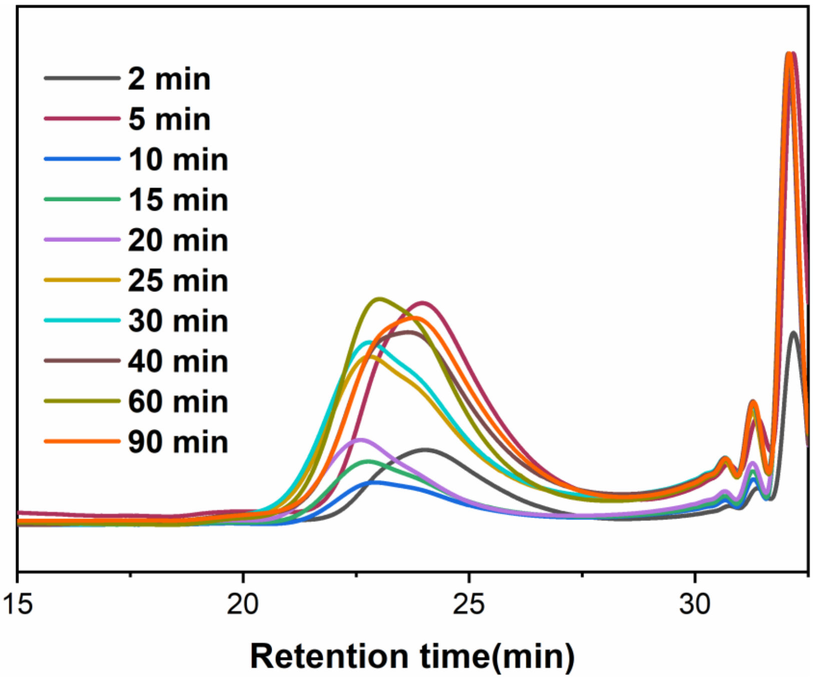

Figure S7. The GPC traces at different reaction times within a [M1]:[TBD] ratio of 1000:1 at 150 ℃.

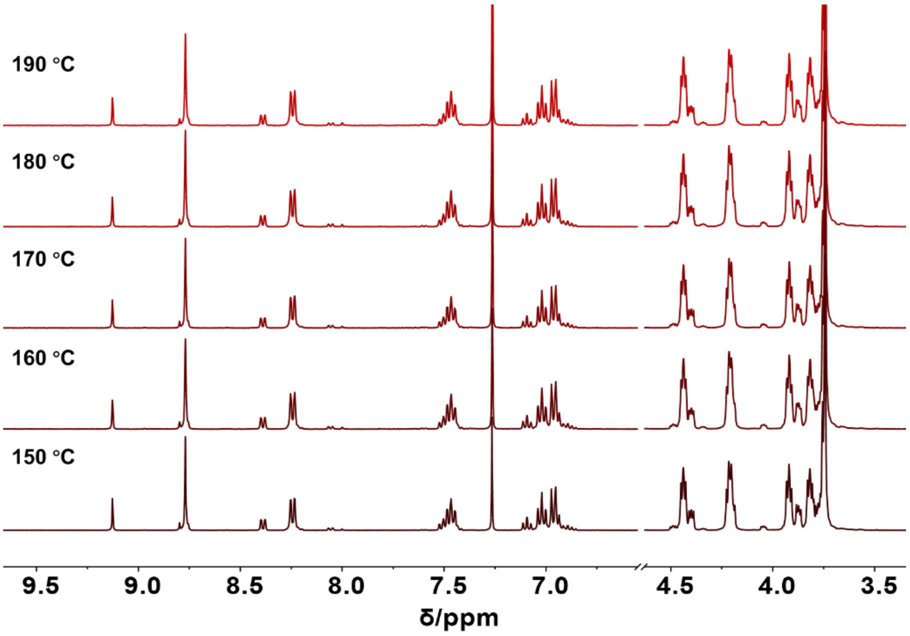

Figure S8. ^{1}H NMR spectra at different temperature within a [M1]:[TBD] ratio of 1000:1 for 1h.

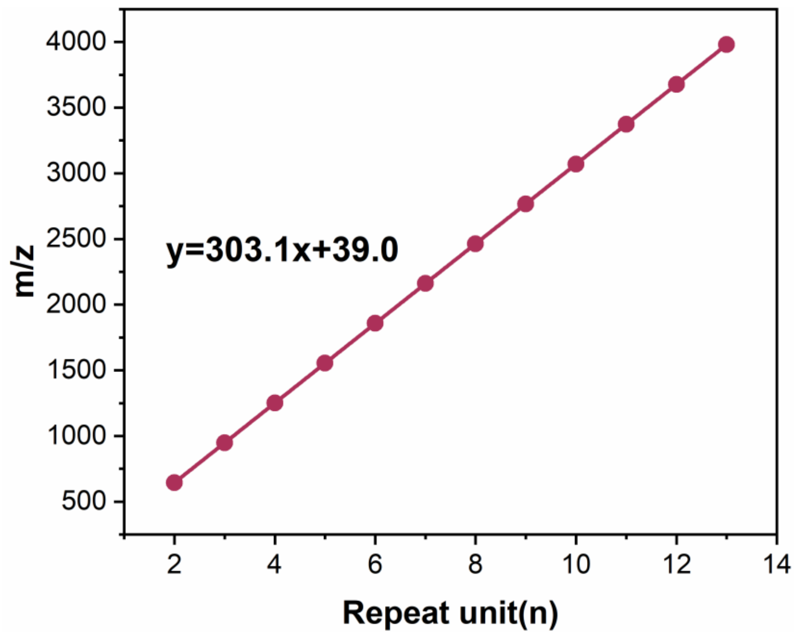

Figure S9. Polt of m/z values (y) of **Poly-M1** versus the theoretical number of repeat units (x) with a slope of 39.

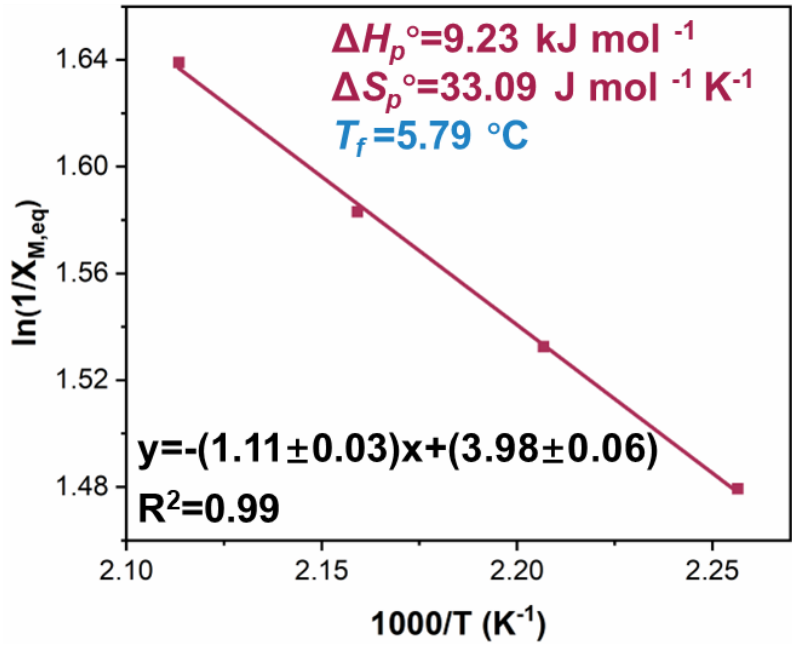

Figure S10. Van't Hoff analysis of the polymerization of **M2** under bulk conditions.

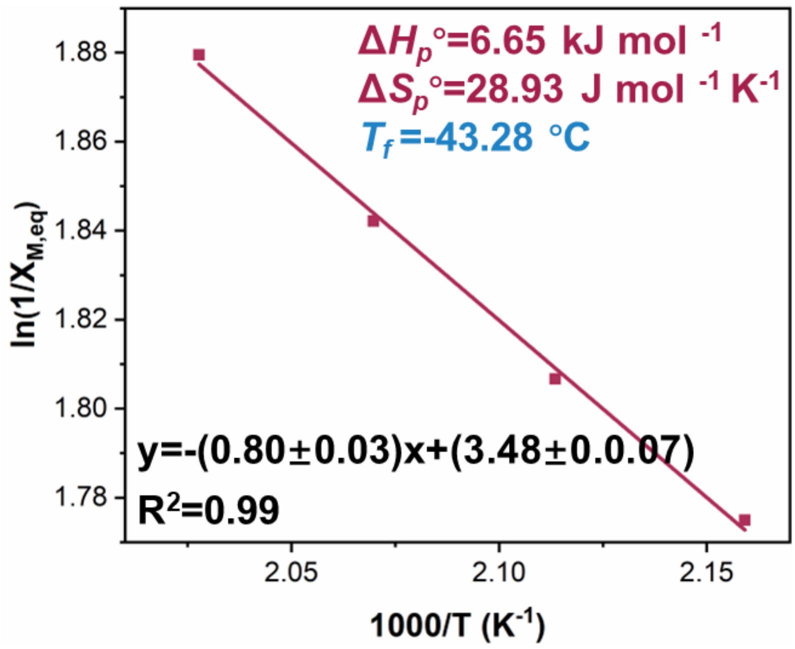

Figure S11. Van't Hoff analysis of the polymerization of **M3** under bulk conditions.

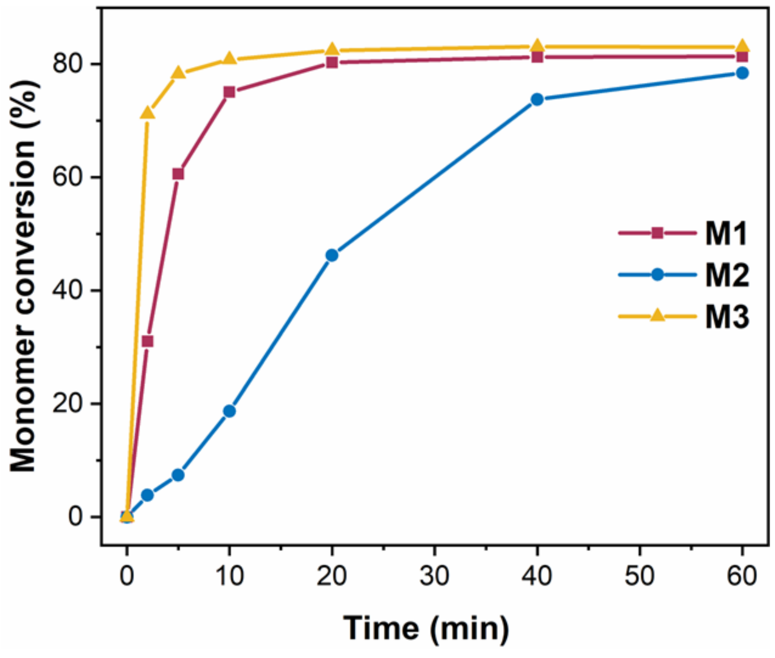

Figure S12. Plots of monomer conversion as a function of reaction time for M1–M3 at 190 ℃.

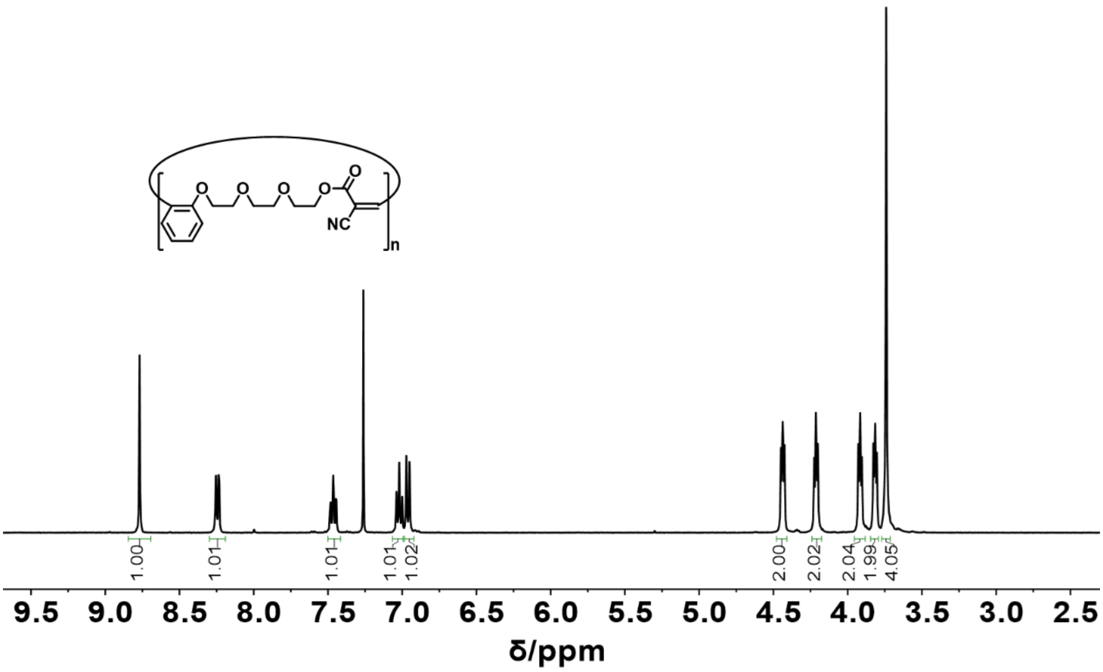

Figure S13.^{1}H NMR spectra of Poly-M1 (Mn=44.1 kDa, *Đ*=1.67).

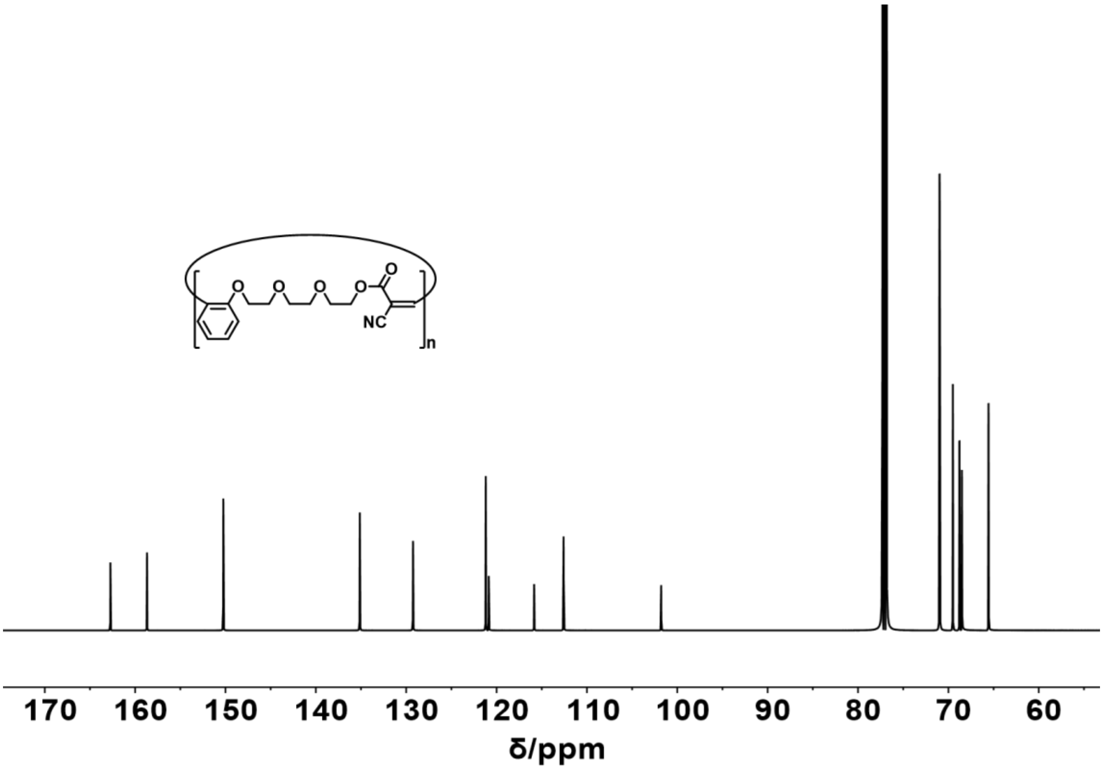

Figure S14.^{13}C NMR spectra of Poly-M1 (Mn=44.1 kDa, *Đ*=1.67).

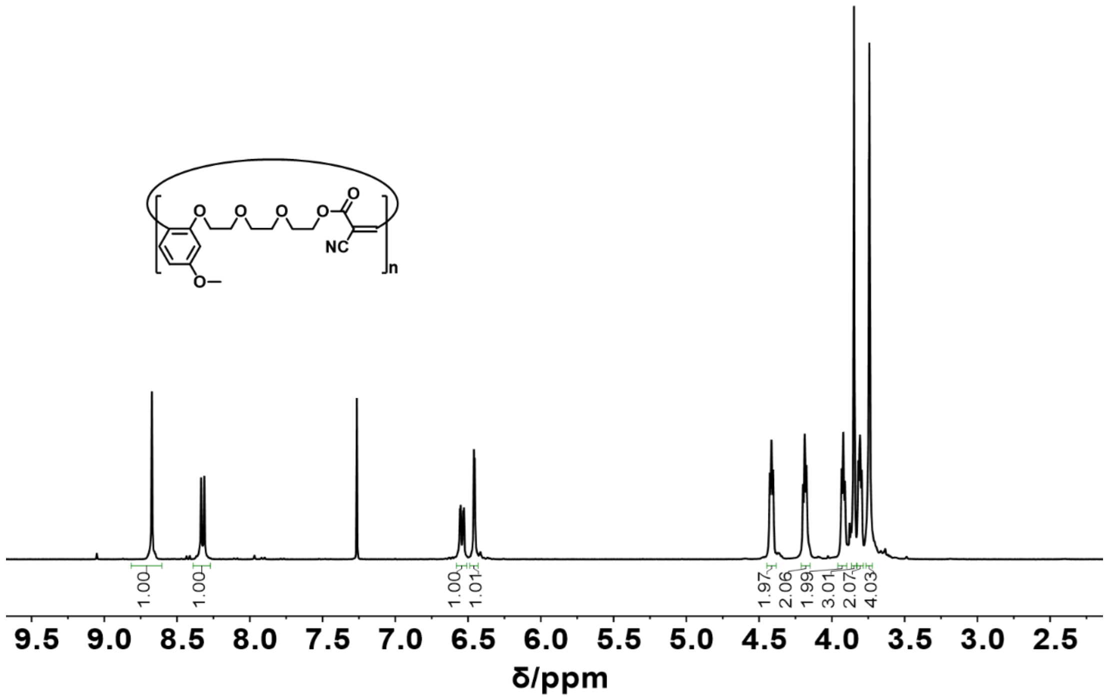

Figure S15.^{1}H NMR spectra of Poly-M2 (Mn=24.3 kDa, *Đ*=1.47).

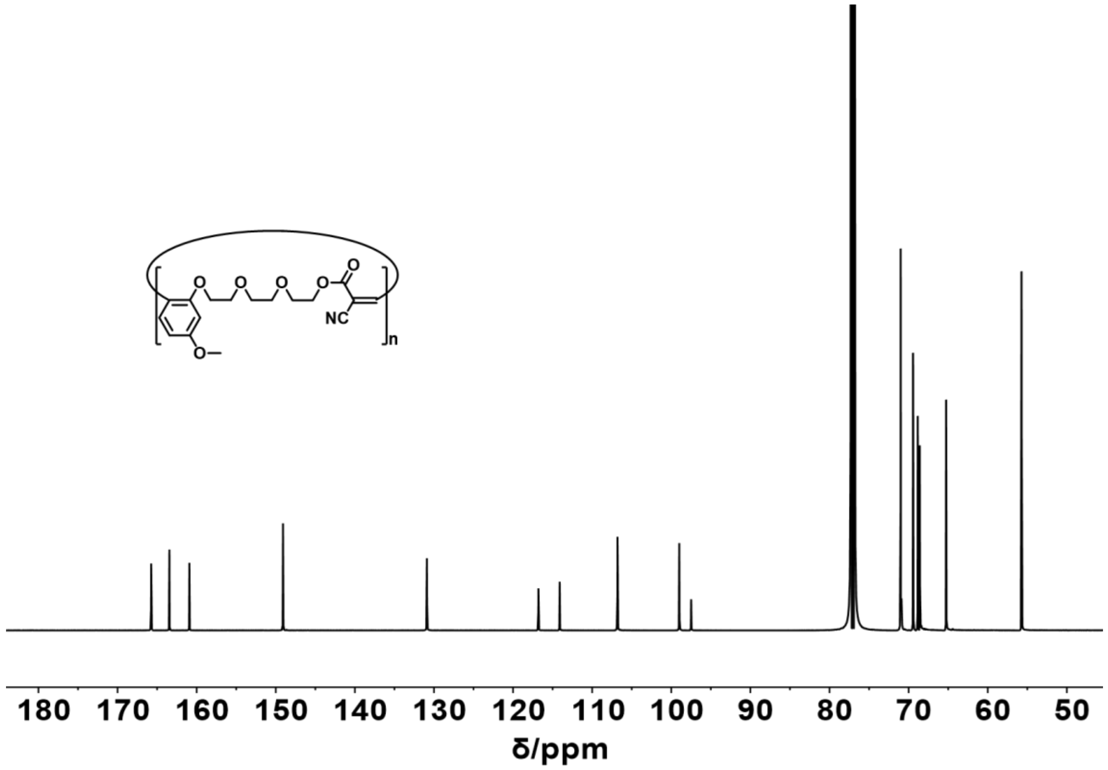

Figure S16.^{13}C NMR spectra of Poly-M2 (Mn=24.3 kDa, *Đ*=1.47).

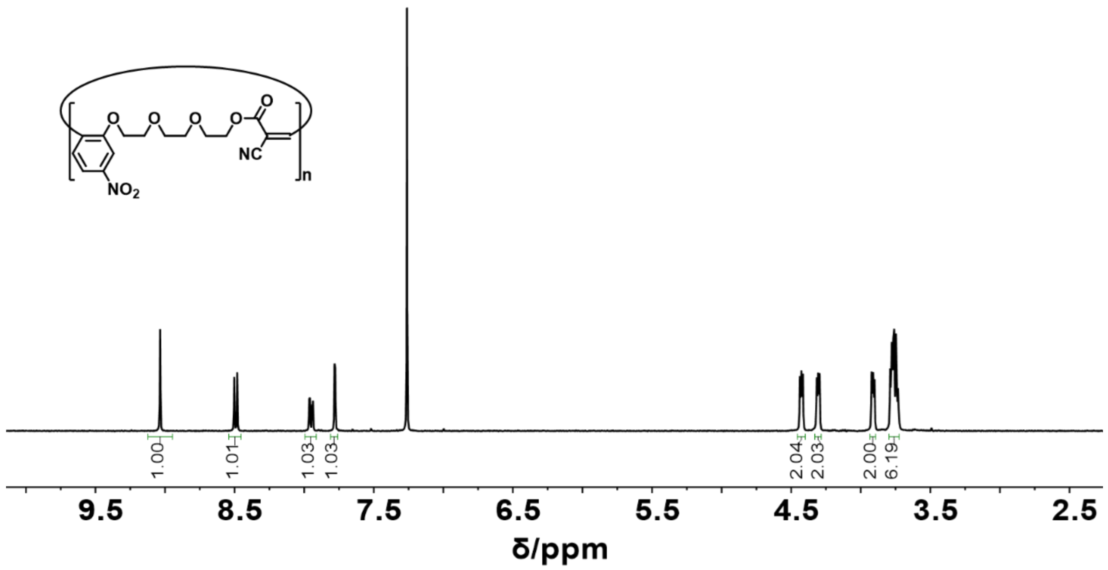

Figure S17.^{1}H NMR spectra of Poly-M3 (Mn=33.3 kDa, *Đ*=1.91).

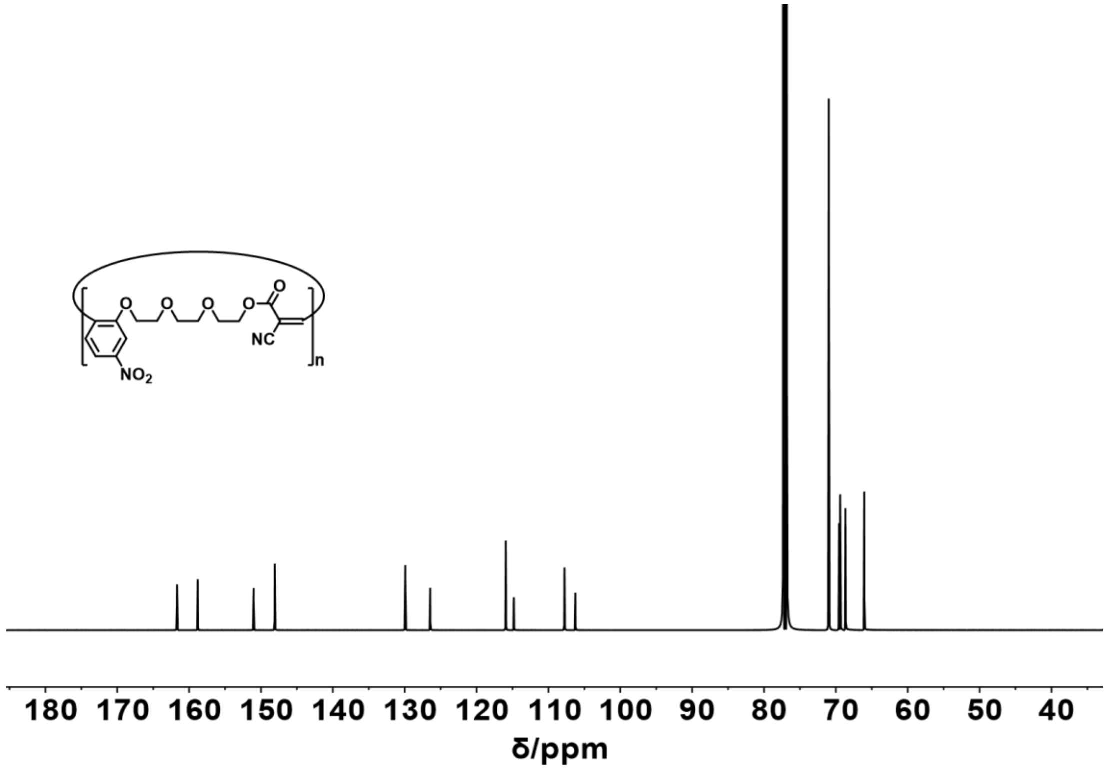

Figure S18.^{13}C NMR spectra of Poly-M3.

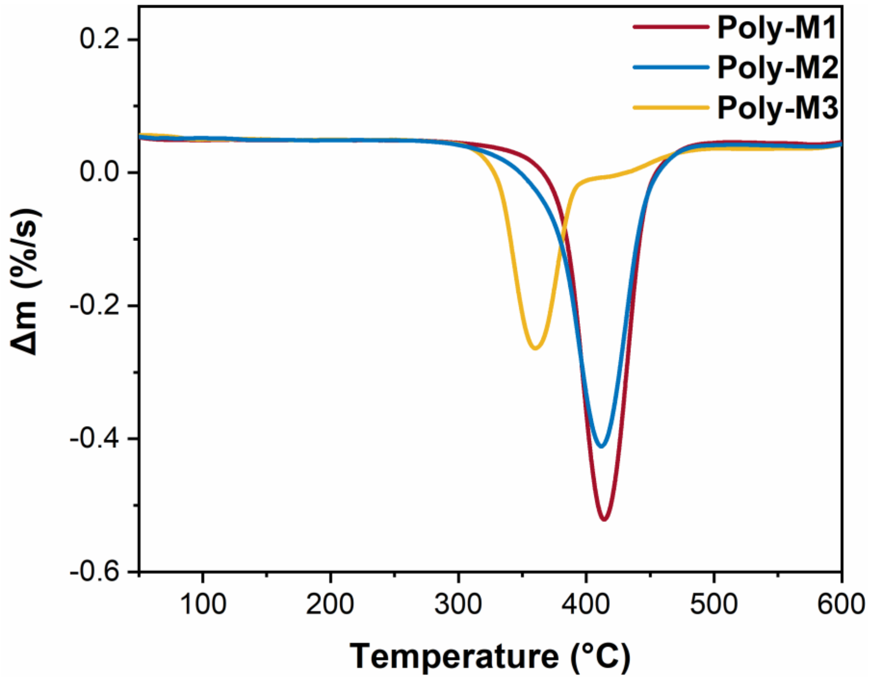

Figure S19. Differential thermogravimetry (DTG) analysis curves of Poly-M1 to Poly-M3.

Table S4. Thermal and mechanical properties of Poly-M1 to Poly-M3.

Sample	T_g (℃)	$T_{d5\%}$ (℃)	T_{max} (℃)	Tensile strength (MPa)	Young’s modulus (MPa)	Elongation at break (%)
Poly-M1	22.6	373.3	414.1	7.92 ± 0.94	308.9 ± 61.0	621.1 ± 86.2
Poly-M2	39.4	359.5	412.2	31.39 ± 2.79	984.2 ± 141.5	3.4 ± 0.2
Poly-M3	49.5	341.9	360.5	39.09 ± 2.66	1780.2 ± 205.9	3.0 ± 0.5

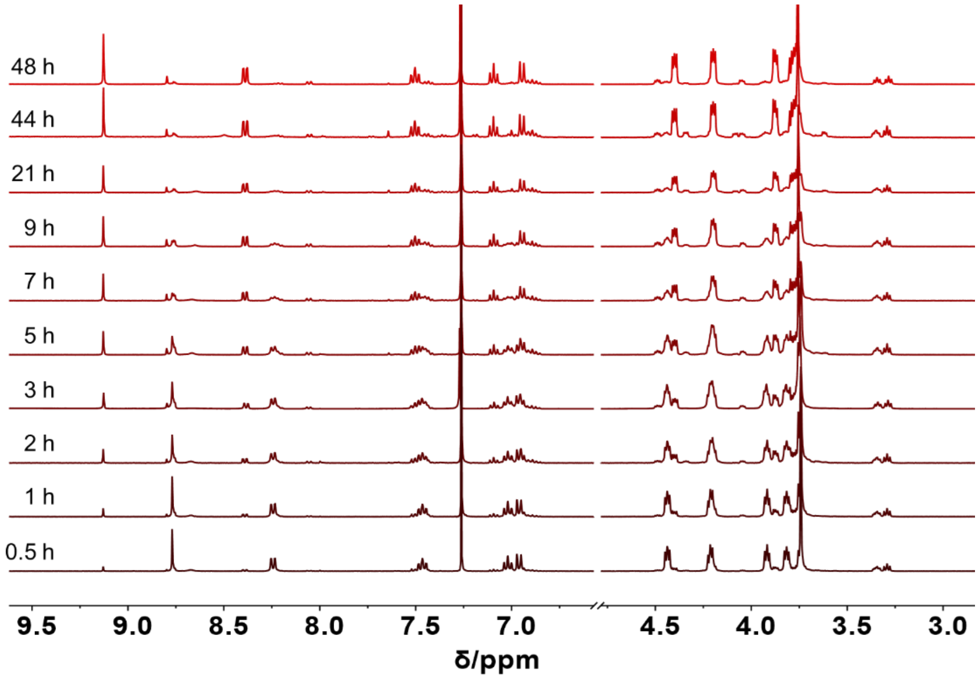

Figure S20. ^{1}H NMR spectra of Poly-M1 after depolymerization for different durations in $CDCl_3$ (100 mM, 10 mol% TBD) at 50 ℃.

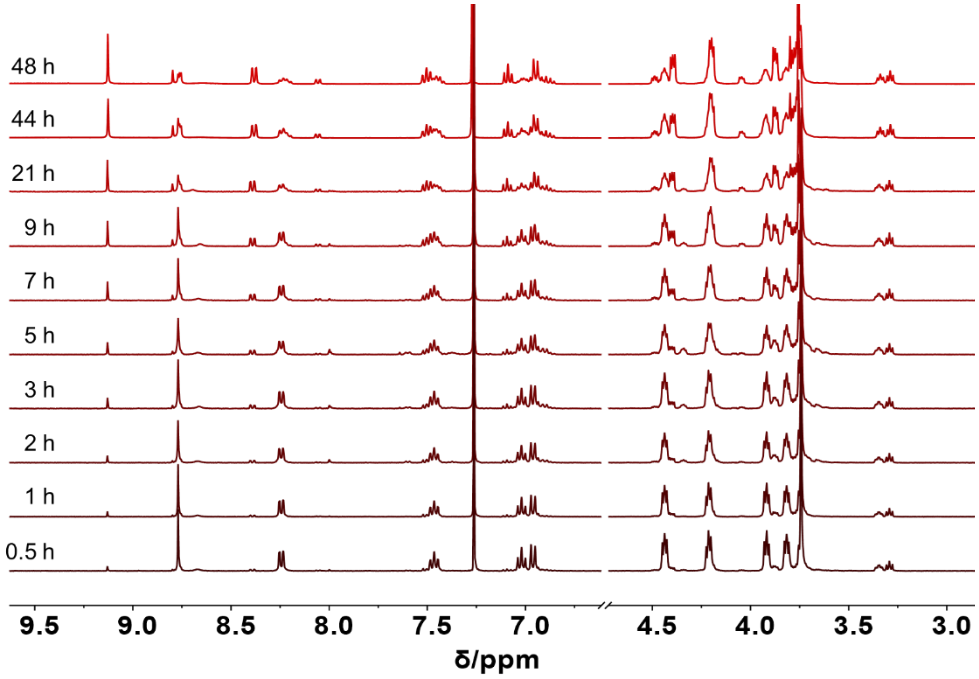

Figure S21. ^{1}H NMR spectra of Poly-M1 after depolymerization for different durations in $CDCl_3$ (100 mM, 10 mol% TBD) at 25 ℃.

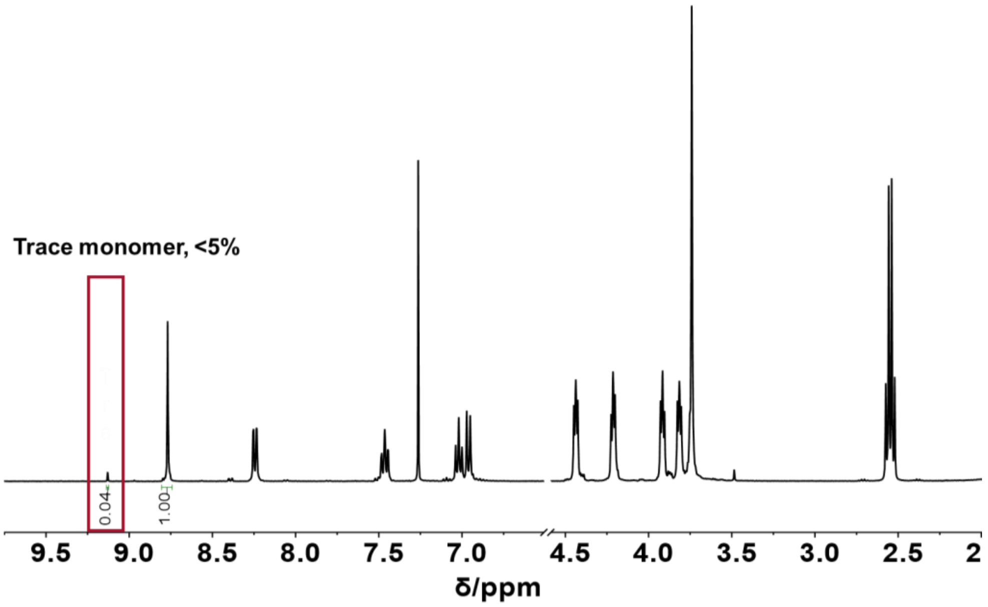

Figure S22. ^{1}H NMR spectra of Poly-M1 after depolymerization for 48 in $CDCl_3$ (100 mM, 1eq TEA) at 50 ℃.

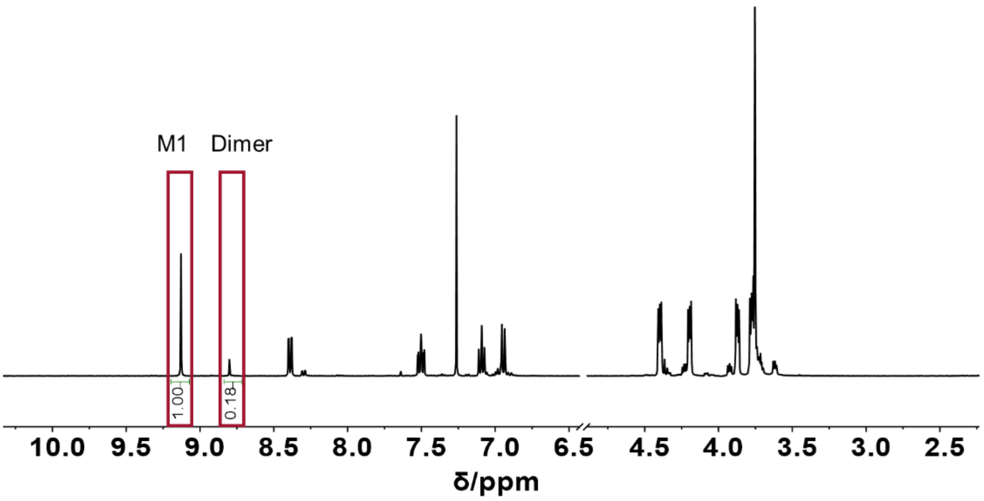

Figure S23. The ^{1}H NMR spectrum of the product obtained after the poly-M1 undergoes depolymerization, followed by simple water washing and drying treatment, with a yield of over 90%.

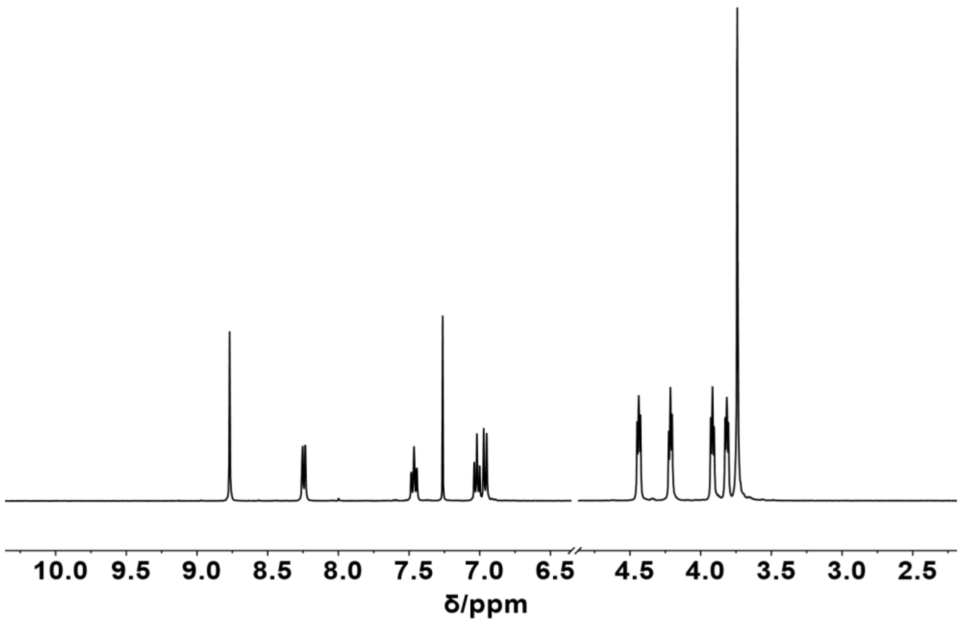

Figure S24. The ^{1}H NMR spectrum of the polymer obtained from the repolymerization of the depolymerization products.

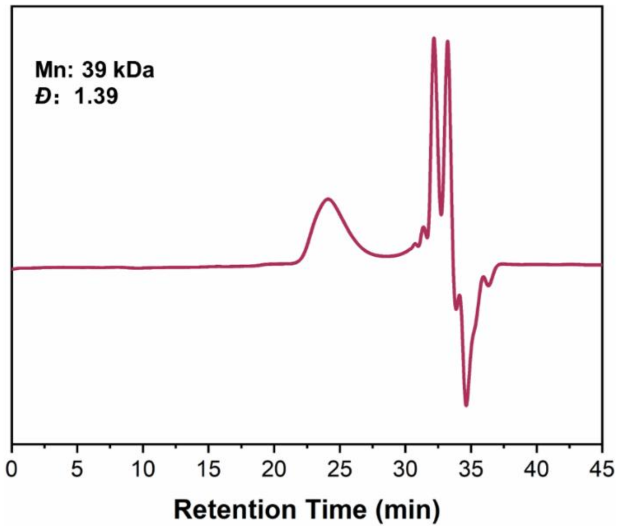

Figure S25. The GPC curve of the depolymerization product after re-polymerization.

Appendix

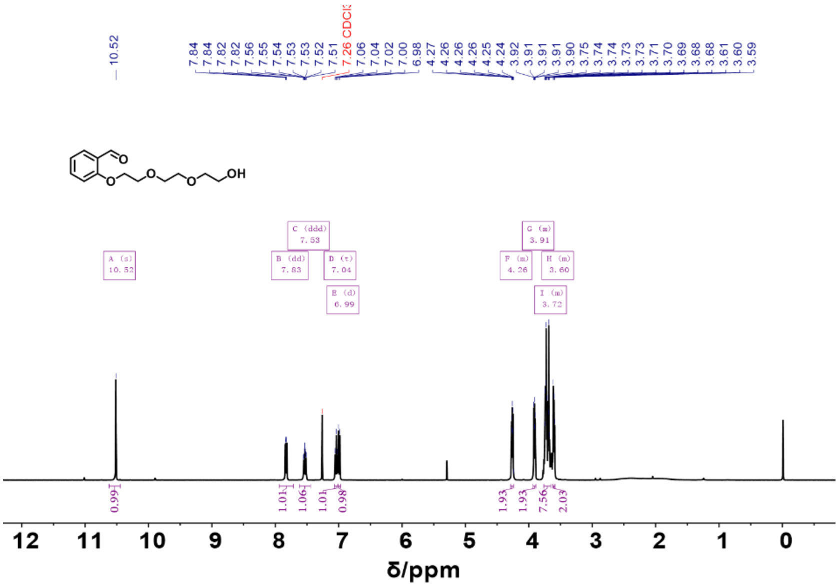

^{1}H NMR (400 MHz, $CDCl_3$,298K) of C1.

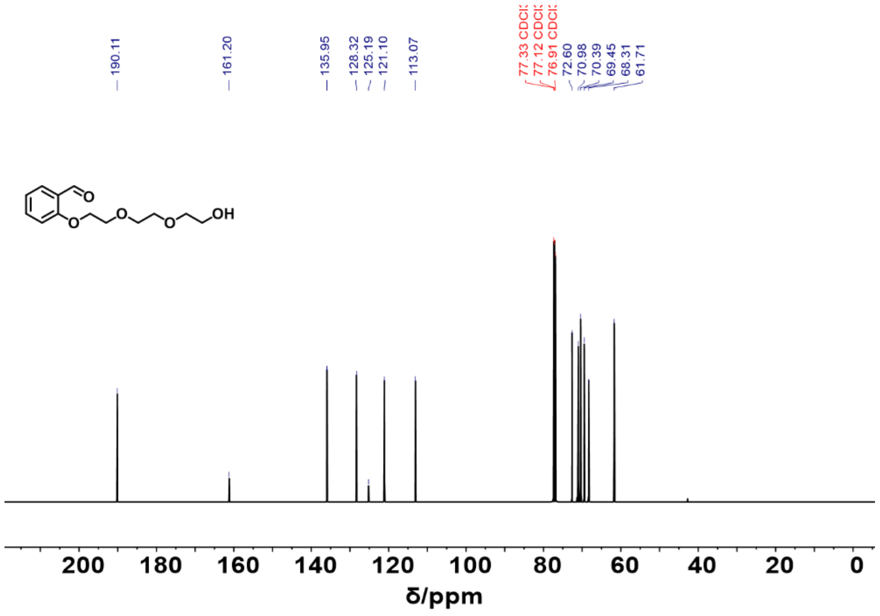

^{13}C NMR (150 MHz, $CDCl_3$,298K) of C1.

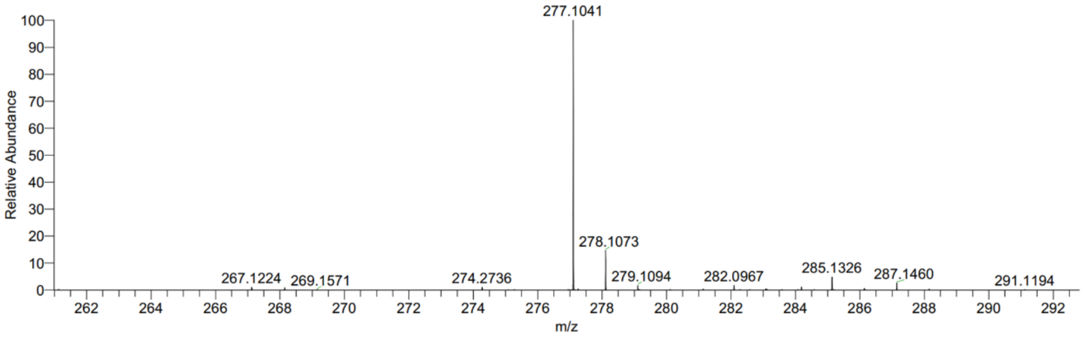

HRSM data of C1([M+Na]$^+$).

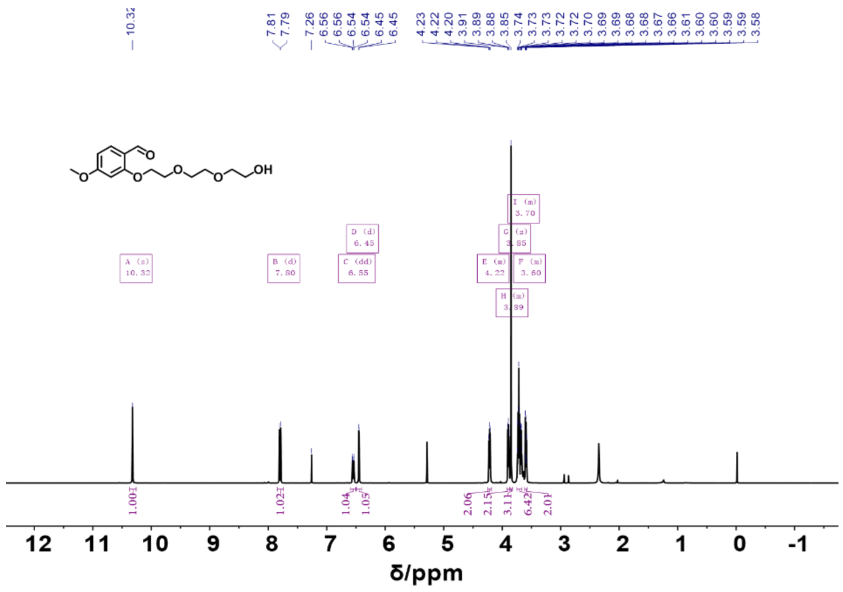

^{1}H NMR (400 MHz, $CDCl_3$,298K) of C2.

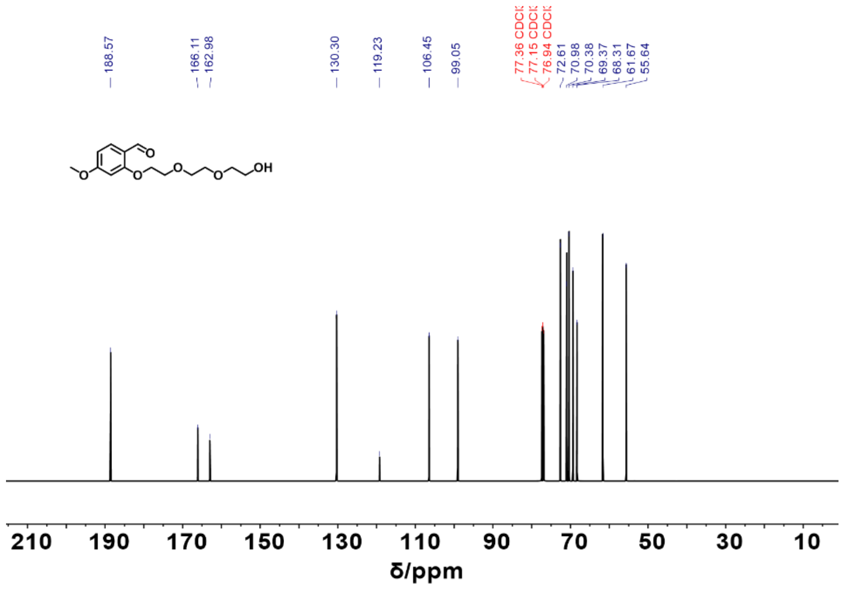

^{13}C NMR (150 MHz, $CDCl_3$,298K) of C2.

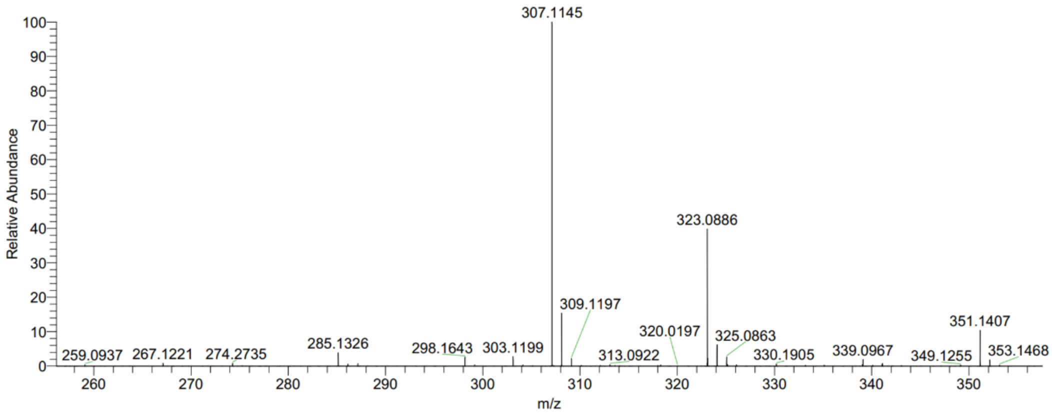

HRSM data of C2([M+Na]$^{+}$).

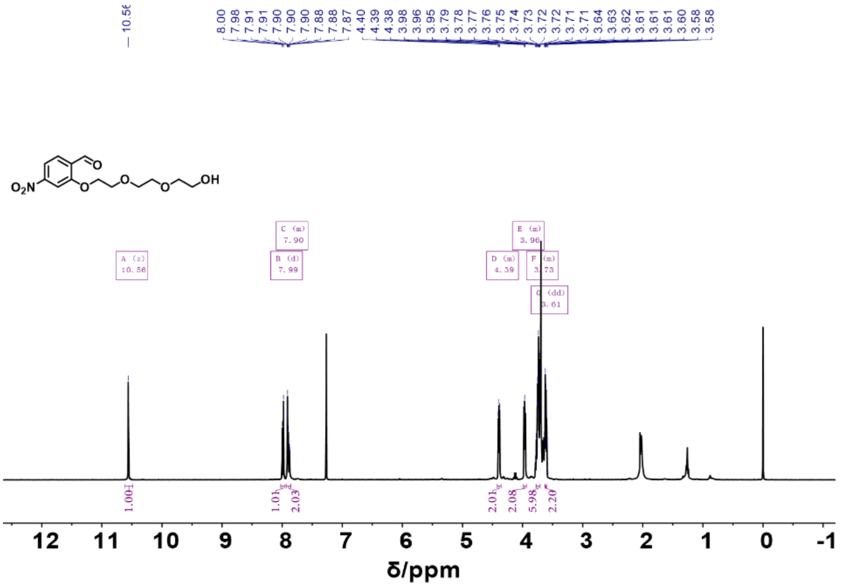

^{1}H NMR (400 MHz, $CDCl_3$,298K) of C3.

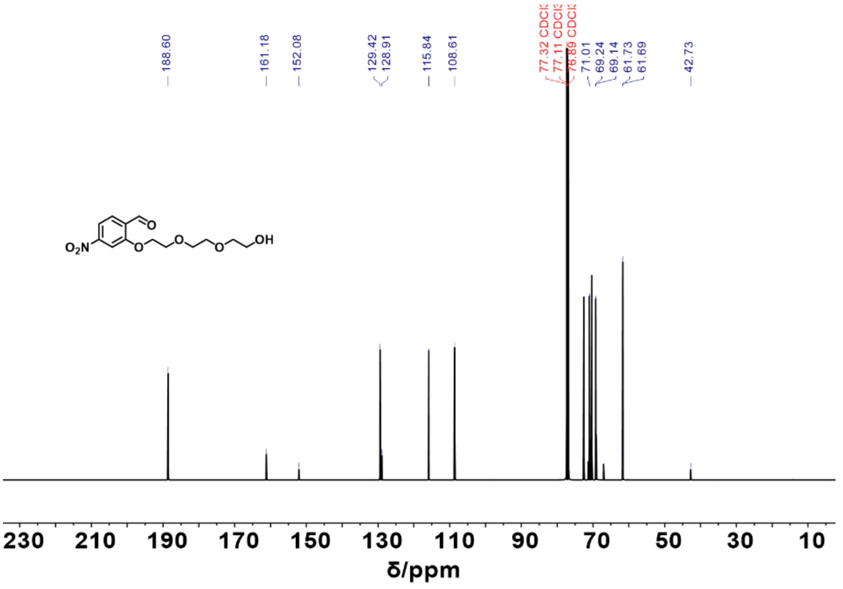

^{13}C NMR (150 MHz, $CDCl_3$,298K) of C3.

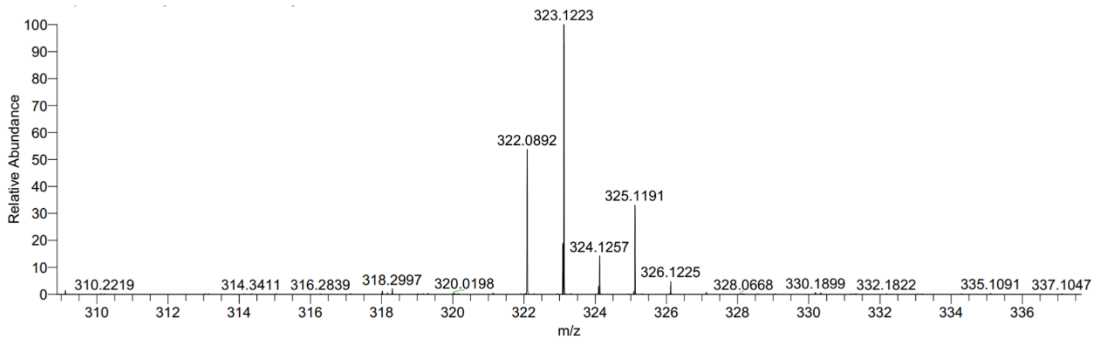

HRSM data of C3([M+Na]$^{+}$).

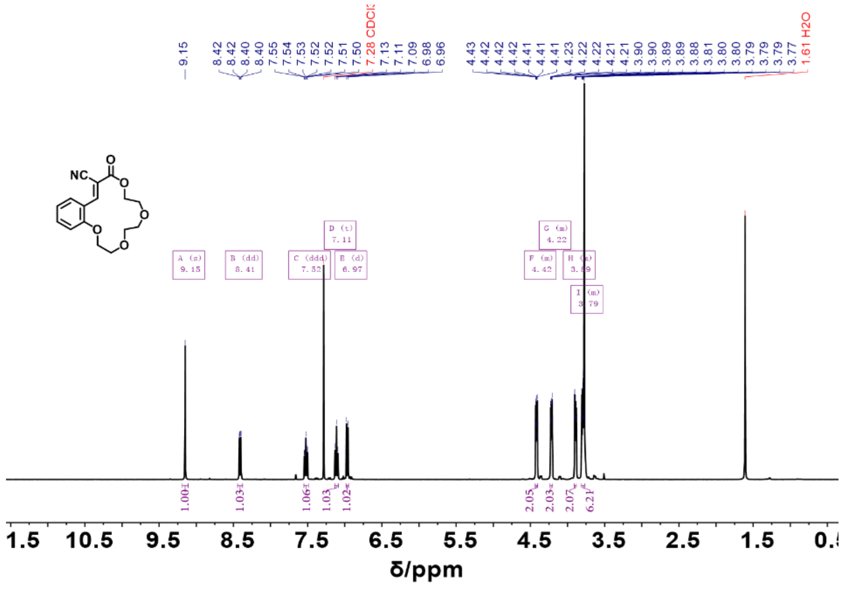

^{1}H NMR (400 MHz, $CDCl_3$,298K) of M1.

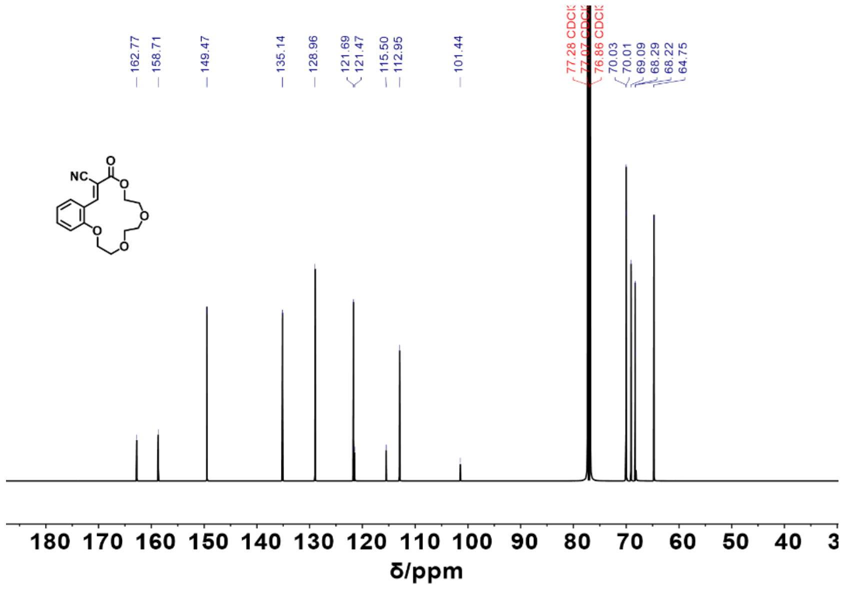

^{13}C NMR (150 MHz, $CDCl_3$,298K) of M1.

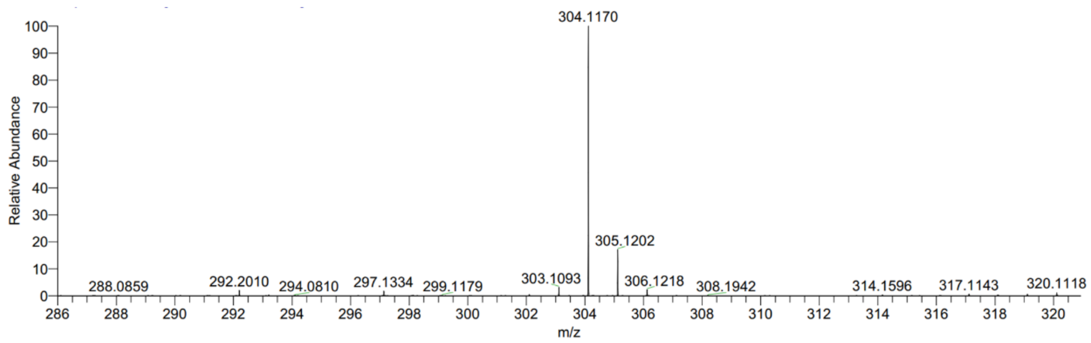

HRSM data of M1([M+H]$^+$).

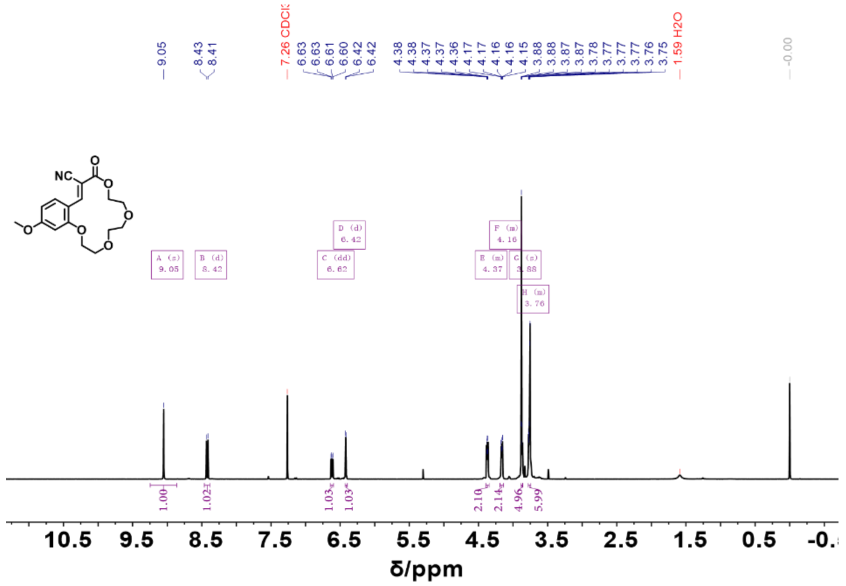

^{1}H NMR (400 MHz, $CDCl_3$,298K) of M2.

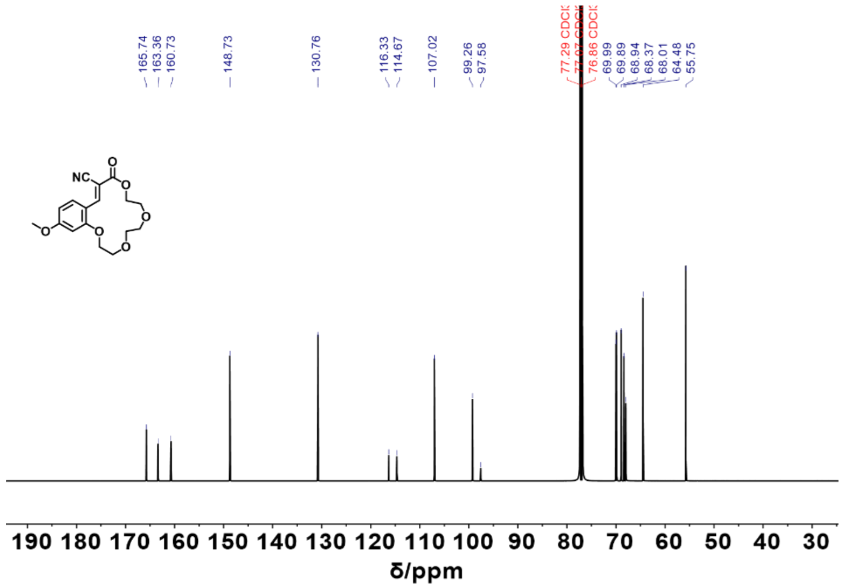

^{13}C NMR (150 MHz, $CDCl_3$,298K) of M2.

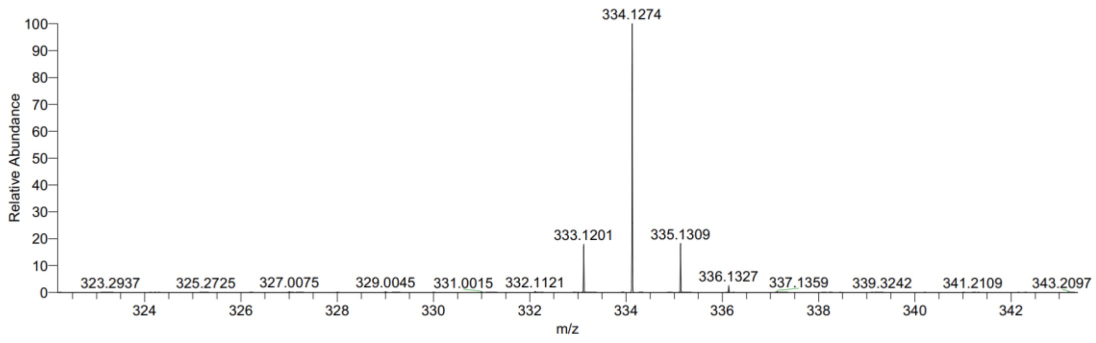

HRSM data of M2([M+H]$^+$).

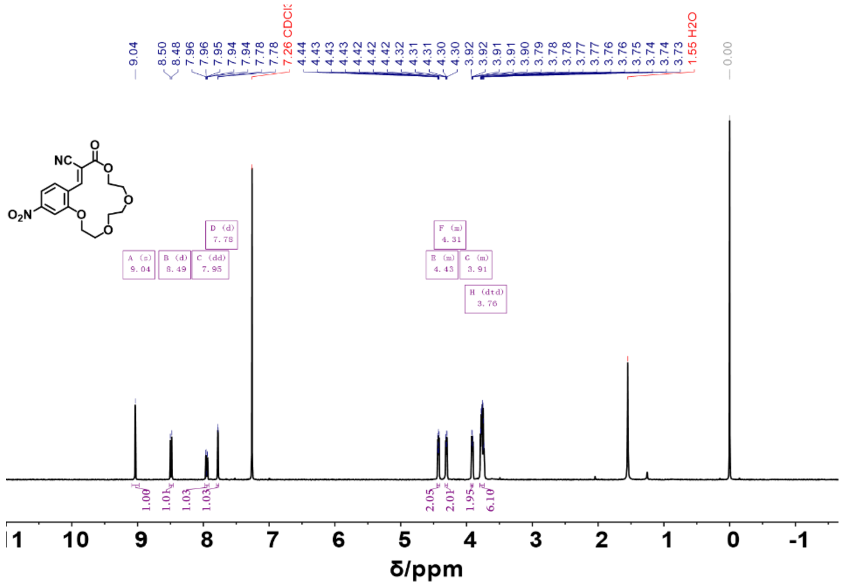

^{1}H NMR (400 MHz, $CDCl_3$,298K) of M3.

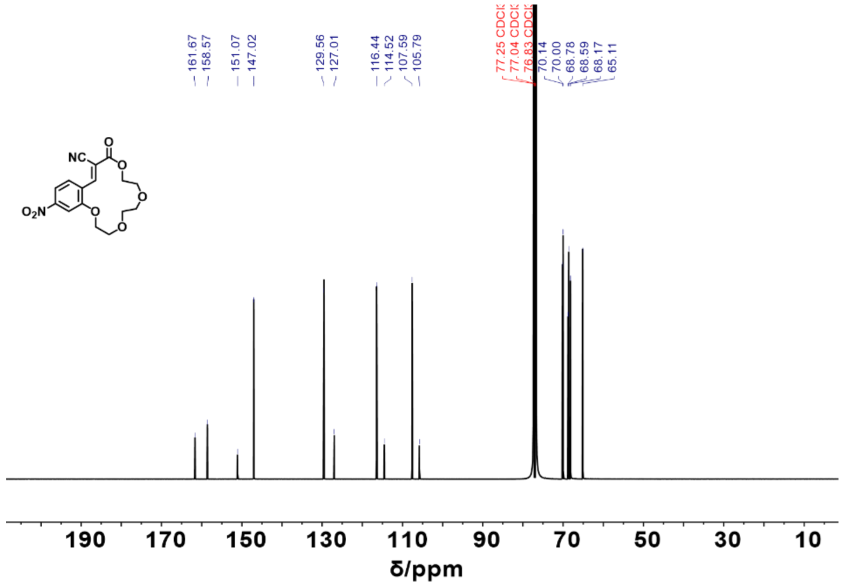

^{13}C NMR (150 MHz, $CDCl_3$,298K) of M3.

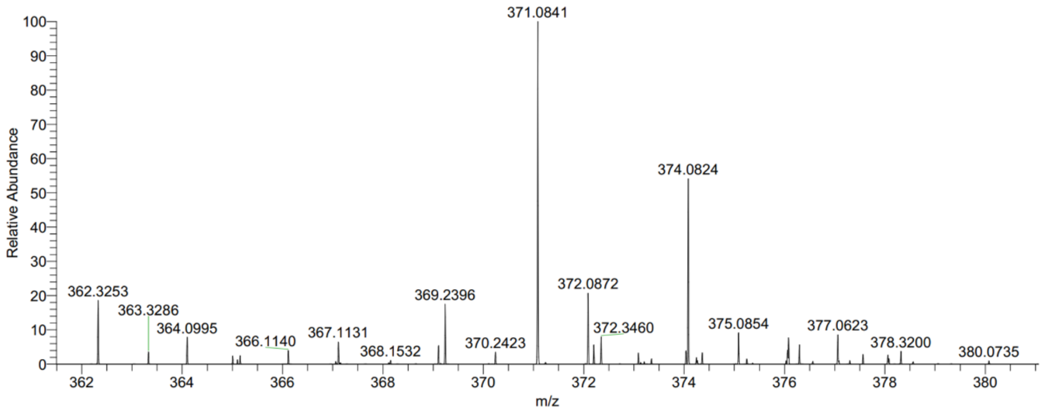

HRSM data of M3($[M+Na]^+$).
